# Cocktail of isobavachalcone and curcumin enhance eradication of *Staphylococcus aureus* biofilm from orthopedic implants by gentamicin and alleviate inflammatory osteolysis

**DOI:** 10.3389/fmicb.2022.958132

**Published:** 2022-09-23

**Authors:** Yan Chen, Hao Hu, Fangli Huang, Zemin Ling, Bolin Chen, Bizhi Tan, Tingxuan Wang, Xiao Liu, Chun Liu, Xuenong Zou

**Affiliations:** ^1^Department of Spine Surgery, The First Affiliated Hospital, Sun Yat-sen University, Guangzhou, China; ^2^Guangdong Provincial Key Laboratory of Orthopaedics and Traumatology, The First Affiliated Hospital, Sun Yat-sen University, Guangzhou, China; ^3^Precision Medicine Institute, The First Affiliated Hospital, Sun Yat-sen University, Guangzhou, China

**Keywords:** biofilm, *Staphylococcus aureus*, synergy, isobavachalcone, curcumin, orthopedic device-related infection, inflammatory osteolysis, myeloid-derived suppressor cells

## Abstract

Orthopedic device-related infection (ODRI) caused by *Staphylococcus aureus*, especially methicillin-resistant *S. aureus* (MRSA) biofilm may lead to persist infection and severe inflammatory osteolysis. Previous studies have demonstrated that both isobavachalcone and curcumin possess antimicrobial activity, recent studies also reveal their antiosteoporosis, anti-inflammation, and immunoregulatory effect. Thus, this study aims to investigate whether the combination of isobavachalcone and curcumin can enhance the anti-*S. aureus* biofilm activity of gentamicin and alleviate inflammatory osteolysis *in vivo*. EUCAST and a standardized MBEC assay were used to verify the synergy between isobavachalcone and curcumin with gentamicin against planktonic *S. aureus* and its biofilm *in vitro*, then the antimicrobial and immunoregulatory effect of cocktail therapy was demonstrated in a femoral ODRI mouse model *in vivo* by μCT analysis, histopathology, quantification of bacteria in bone and myeloid-derived suppressor cell (MDSC) in bone marrow. We tested on standard MSSA ATCC25923 and MRSA USA300, 5 clinical isolated MSSA, and 2 clinical isolated MRSA strains and found that gentamicin with curcumin (62.5–250 μg/ml) and gentamicin with isobavachalcone (1.56 μg/ml) are synergistic against planktonic MSSA, while gentamicin (128 μg/ml) with curcumin (31.25–62.5, 250–500 μg/ml) and gentamicin (64–128 μg/ml) with isobavachalcone (1.56–12.5 μg/ml) exhibit synergistic effect against MSSA biofilm. Results of further study revealed that cocktail of 128 μg/ml gentamicin together with 125 μg/ml curcumin +6.25 μg/ml isobavachalcone showed promising biofilm eradication effect with synergy against USA300 biofilm *in vitro*. Daily intraperitoneal administration of 20 mg/kg/day isobavachalcone, 20 mg/kg/day curcumin, and 20 mg/kg/day gentamicin, can reduce inflammatory osteolysis and maintain microarchitecture of trabecular bone during orthopedic device-related MRSA infection in mice. Cocktail therapy also enhanced reduction of MDSC M1 polarization in peri-implant tissue, suppression of MDSC amplification in bone marrow, and Eradication of USA300 biofilm *in vivo*. Together, these results suggest that the combination of isobavachalcone and curcumin as adjuvants administrated together with gentamicin significantly enhances its antimicrobial effect against *S. aureus* biofilm, and can also modify topical inflammation in ODRI and protect bone microstructure *in vivo*, which may serve as a potential treatment strategy, especially for *S. aureus* induced ODRI.

## Introduction

Device-related Infection (DRI; Costerton et al., 1999) is a serious complication of orthopedic surgery, which may lead to severe bacterial osteomyelitis (OM) and inflammatory osteolysis and the destruction of surrounding soft tissues (Molin and Tolker-Nielsen, 2003; Arciola et al., 2008). Despite the improvement of aseptic surgery techniques and the standardized use of antimicrobial drugs over a considerable period of time, orthopedic device-related infections still have an incidence of ~2.3%–20% (depending on the patient’s systemic status and surgical area conditions, as well as the type of surgery and implant; Ribeiro et al., 2012; Oikonomidis et al., 2020). The infection incidence ranges from 1% to 2% in first total joint replacement and 2% to 6% in revision joints (Rao et al., 2011; Fagotti et al., 2018), while the incidence of infection after spinal internal fixation fusion ranges from 0.7% to 11.9% (Schimmel et al., 2010). With the increased use of orthopedic implants, including various types of joint prostheses, plates, and screws, the number of implant-related infections will likely continue to increase. DRI and the resulting osteomyelitis are usually caused by *Staphylococcus aureus* (*S. aureus*), which, as a Gram-positive (G+) bacterium (Montanaro et al., 2011), is also the main pathogen causing community-associated hospital-acquired infections (Jones et al., 2014). However, with the overuse of antibiotics, methicillin-resistant *S. aureus* (MRSA) has gradually increased in the proportion of clinically isolated *S. aureus* (Hiramatsu et al., 2002), showing a > 50% prevalence of nosocomial infections in several Asian countries (Drago et al., 2007).

Bacterial biofilm (BF) is an important mechanism mediating the antibiotic resistance of MRSA, which is a multilayered three-dimensional complex structure formed by bacteria as well as hydrated extracellular polymers (EPS) composed of polysaccharides, proteins, lipids and extracellular nucleic acids (Flemming et al., 2007). The formation of biofilm on the surface of implants creates a physical barrier that makes it difficult for antibiotics to enter, while the microenvironment within the biofilm, such as pH and ion concentration, is significantly altered, thus reducing the effectiveness of antibiotics. Strategies to cope with biofilm-induced resistance include developing novel antibiotics, increasing antibiotics dosing, and the combination of multiple antibiotics or small molecules. However, the speed of new antibiotic development is limited, and high concentrations of antibiotics or multiple antibiotic combinations may lead to liver and kidney damage(Lebeaux et al., 2014; Huang et al., 2020); therefore, screening small molecules to be used in combination with existing antibiotics to enhance their efficacy is a very economical and practical strategy.

Previous studies have shown that many molecules from widely used herbal medicines have antibacterial effect. Curcumin (CRM), a natural polyphenolic substance, was first isolated and identified in the rhizome of the turmeric, and also as one of the components in the patent Chinese traditional medicine compound Xian-ling-gu-bao (XLGB), which was approved by China’s CFDA in the treatment of osteoporosis, aseptic osteolysis, fracture, and osteoarthritis. Several studies have shown that CRM has potential antioxidant, anti-inflammatory, antibacterial and antitumor effects (Daniel et al., 2004; Moghadamtousi et al., 2014; Bimonte et al., 2016; Kunnumakkara et al., 2017). In terms of antibacterial activity, curcumin increases the bidirectional permeability of the cell membrane of *S. aureus* (Tyagi et al., 2015) and its antimicrobial activity may act by disrupting cell membrane (Mun et al., 2014; Teow et al., 2016), while other studies have shown enhanced membrane penetration and membrane depolarization by its improved molecules (Prince et al., 2019). Isobavachalcone (ISB) is a pentenyl chalcone isolated from plants of the Fabaceae, Umbelliferae, Moraceae, Schisandrae, and Bee families, which is also a component of XLGB. ISB is active against Gram-positive bacteria, mainly methicillin-sensitive *Staphylococcus aureus* (MSSA) and methicillin-resistant *Staphylococcus aureus* (MRSA). It’s reported that ISB inhibited more than 50% of MSSA and MRSA biofilm formation at 0.78 μg/ml, while cytotoxicity assays showed that it did not influence cell viability (Wang X. et al., 2021). Curcumin was widely used for decade and served as a FDA-approved compounded drug additives for oral administration, and for injection in some circumstances, and they both have antimicrobial effects alone. Thus, whether they have synergistic effects in combination with conventional antibiotics that increase their antimicrobial effects is worthy of in-depth study.

In addition, it has been reported that following orthopedic device-related infection, *S. aureus* biofilm could induce polarization of macrophages in the surrounding bone marrow lumen toward pro-inflammation M1 macrophages. M1-type macrophages are associated with an excessive and sustained activation of the inflammatory response, which may lead to inflammatory-related bone resorption and consequent implant loosening. Interestingly, it has been shown that curcumin could inhibit M1-type macrophage polarization to prevent osteocyte apoptosis in a glucocorticoid-related femoral head necrosis mouse model (Jin et al., 2020). Another study showed that curcumin could maintain the M0-like phenotype of macrophage under the influence of PE particles and inhibit macrophage-involved osteolysis and inflammation *via* promoting cholesterol efflux(Liu et al., 2019). Besides, Isobavachalcone could prevent osteoporosis by inhibiting M1 polarization of macrophages (Wang X. et al., 2021). Therefore, curcumin and Isobavachalcone may prevent inflammatory bone resorption associated with *S. aureus* infection by modulating the macrophage phenotype.

In summary, curcumin and Isobavachalcone have both anti-bacterial and anti-inflammatory effects; however, it remains unclear whether they have synergistic effects with conventional antibiotics and whether their combination exerts stronger effects on preventing inflammatory bone resorption in orthopedic device-related infection. In order to address the above questions, this study would first verify the synergistic effects of curcumin and Isobavachalcone separately or simultaneously with conventional antibiotics through *in vitro Staphylococcus aureus* biofilm model, and then confirm the anti-infective and anti-inflammatory effect *in vivo* through an MRSA-induced mouse femoral implant infection model.

## Materials and methods

### Microbiology

#### Bacterial strains and reagents

Methicillin-susceptible *S. aureus* (MSSA) ATCC 25923 and Methicillin-resistant *S. aureus* (MRSA) ATCC 700699 (USA300) were purchased from China General Microbiological Culture Collection Center (CGMCC), China. Clinical isolated strain *S. aureus* JAR was provided by AO Research Institute Davos, Switzerland. Clinical isolated strains *S. aureus* MSSA 2222, MSSA 2557, MSSA 2039, MSSA 2031, MRSA 2435, and MRSA 2027 from various patients with ODRI were provided by The First Affiliated Hospital of Jinan University, China. Gentamicin sulfate (HY-A0276), Daptomycin (HY-B0108), Rifampicin (HY-B0272), and Levofloxacin (HY-B0330) were purchased from MedChemExpress, China. Curcumin (HY-N0005) and isobavachalcone (HY-13065) were purchased from MedChemExpress, China. All reagents were prepared as stock solutions according to manufacturer’s instructions and stored at −20°C.

#### Bacteria suspensions and biofilm cultural conditions

Isolated colonies of *S. aureus* strains on TSA agar plate were picked to inoculate a flask containing 40 ml of Tryptone Soya Broth (TSB, 1268857, OXOID) and incubated on a shaker at 100 rpm at 36 ± 1°C overnight to obtain bacteria suspension. The overnight culture was used for the inoculum on MBEC™ Biofilm Inoculator (16120015, Innovotech, Canada) according to manufacturer instructions to generate biofilm. Bacteria suspensions were centrifuged and washed with PBS twice, then resuspended in PBS, and sonicated for 3 min. The bacterial suspension was adjusted to an OD_600nm_ range between 1.1 and 1.2 (~4–5 × 10^8^ per ml). Vortexed again, diluted 22 μl of the bacteria solution with 22 ml TSB (with 1% human plasma, P9523, Sigma, China) in the trough base to reach a 1,000× dilution (~4–5 × 10^8^ per ml). Then, the peg lid was placed on the trough base. The inoculator was placed on a rocking table set to 5 rocks/min at 36 ± 1°C for 24 h. The plates’ orientation was changed for 90° every 12 h. Medium for each plate was refreshed every day. For biofilms used for laser scanning confocal microscopy imaging, 24-well cell slides were used instead of MBEC inoculators, and the rest of the culture conditions remain the same.

#### MIC and MBC against *Staphylococcus aureus*

The minimum inhibitory concentration (MIC) and minimum bactericidal concentration (MBC) of isobavachalcone, curcumin, and antibiotics and their combinations were determined by EUCAST guidelines with modifications for a broth microdilution checkerboard procedure (Kahlmeter et al., 2006; Cusack et al., 2019). The combinations of XLGB molecules and antibiotics on a two-dimensional checkerboard with two-fold dilutions. Checkerboard challenge plates were established using 96-well microtiter plates. The final concentrations of ISB (0.20–12.50 μg/ml) and CRM (0.24–2,000 μg/ml) were chosen according to literature (Yin et al., 2004; Tajbakhsh et al., 2008; Dzoyem et al., 2013; Mun et al., 2013; Sasidharan et al., 2014; Cui et al., 2015; Teow and Ali, 2015; Gunes et al., 2016; Sandikci et al., 2016; Wang et al., 2016; Faegheh et al., 2018), and the antibiotics tested were chosen according to EUCAST database.

Bacteria inoculum adjusted to OD between 1.1 and 1.2 (4–5 × 10^8^/ml) were prepared as aforementioned. Adjust the density of the bacteria suspension to 1–1.25 × 10^7^/ml. 5 μl of 1–1.25 × 10^7^/ml bacteria suspension was inoculated to each well containing 200 μl TSB to reach an approximate density of 5 × 10^5^/ml except for sterile control. OD_600nm_ before incubation were measured, all microtiter plates are incubated at 36.5 ± 1°C for 24 h in an incubator with constant humidity, then microtiter plates were measured at OD_600nm_ again, ratio of OD_600nm_ readings after incubation, and the ones before incubation need to be calculated. In this study, not all solutions were clear and transparent, therefore MIC was defined by MIC_90_ (inhibits 90% of growth). The MIC of antibiotics, ISB, and CRM alone are determined as MIC_antibiotic_, MIC_ISB_, and MIC_CRM_, respectively. The MICs of antibiotics, ISB, and CRM in each combination are also determined as MIC_antibiotic_-combination, MIC_ISB_-combination, and MIC_CRM_-combination, respectively. After MIC was determined, 20 μl of liquid in each well was transferred from microtiter plates to single-well MHA plates by using a 96 solid Pin MULTI-BLOT™ Replicator (V.P. Scientific Inc., United States, VP 405). Colonies on single-well MHA plates were photographed and checked after incubated at 36.5 ± 1°C for 24 h. MBC was defined as the lowest concentration of eradicating bacteria, leading to no colony formation. The MBCs of ISB, CRM, and antibiotics alone are determined as MBC_ISO_, MBC_CRM_, and MBC_antibiotic_, respectively. The MBCs of ISB, CRM, and antibiotics in each combination are also determined as MBC_ISB_-combination, MBC_CRM_-combination, and MBC_antibiotic_-combination, respectively. All modified EUCASTs were repeated at least three times.

#### MBEC assay against *Staphylococcus aureus* biofilm

The minimum biofilm eradication concentration (MBEC) is determined by MBEC™ Assay as per the manufacturer’s instruction with optimized protocol. Checkerboard technique similar to the EUCAST was used to prepare antibiotic challenge plates for MBEC, including a two-fold concentrations gradient both with antibiotics ISB, and CRM according to the results of EUCAST. Inoculum preparation biofilm cultural conditions were described aforementioned. Transfer the MBEC Assay inoculator lid with homogeneous 24 h biofilm to the challenge plate and incubate at 36.5 ± 1°C for 24 h. Rinse biofilms with 96-well plates with 250 μl/well for ~10 s. Detached pegs with sterilized pliers into individual sterile glass vials with 10 ml of D/E broth, vortex for 10 s and sonicate for 15 min to remove the biofilm from the pegs. Vortex the D/E broth again for 3 s before pipetting 200 μl per well into a new 96-well microtiter plate according to the challenge plate layout. All vials with D/E broth will be stored at 4°C in case for further culture. Use a sterile 96 solid Pin Replicator to transfer 20 μl D/E broth in each well onto a single well TSA plate. Check the colony formation on TSA plates after being incubated at 36.5 ± 1°C for 24 h, the desired group of D/E broth with biofilm will be further conducted bacterial cell counts by using a serial dilution plating method. MBEC is defined as the minimum concentration of antimicrobial that eradicates the biofilm. All biofilm experiments were repeated at least three times.

#### Interpretation of EUCAST and MBEC assay results

For the calculation of the Fractional inhibition concentration index (FICI) and Fractional bactericidal concentration index (FBCI), The FICI and FBCI were calculated as follows: FICI = MIC_A_-combined/MIC_A_  + MIC_B_-combined/MIC_B_; FBCI = MBC_A_-combined/ MBC_A_ + MBC_B_-combined/MBC_B_, A and B stand for different antibacterial molecules. The FICI and FBCI data were interpreted the following way: FICI ≤ 0.5 = synergy, FICI > 0.5–4 = no interaction, and FICI > 4.0 = antagonism (Odds, 2003).

After the residual biofilm was quantified, the synergistic effect could not be further calculated based on its MBEC if multiple drug combinations failed to completely remove the biofilm. The commonly used Coefficient of drug interaction (CDI) was introduced to evaluate synergy according to previous studies (Tallarida, 2011; Song et al., 2020), and the calculation is as follows: CDI = AB/(A × B). Based on the residual CFU per peg of each group, AB is the ratio of the combination group to the control group. A or B is the ratio of the stand-alone antibiotics or ISB/CRM group to the control group. Thus, CDI < 1, =1, or >1 indicate drug synergy, superposition or antagonism, respectively, where CDI < 0.7 indicates significant synergy.

#### Fluorescence and confocal imaging of biofilms

MRSA USA300 biofilms were cultured and challenge as cultural conditions detailed previously on sterile cell slides. For live and dead staining, an aliquot (2 ml) of MHB diluted overnight culture was used to incubate the biofilm on cell slides in 12-well plates for 24 h. The 24 h culture biofilm was removed and washed with PBS. Controls were treated with 128 μg/ml of GEN (MBEC90) only, and 128 μg/ml of GEN combined with 125 μm/ml CRM, 6.25 μm/ml ISB, and 125 μm/ml CRM + 6.25 μm/ml ISB, respectively, for 24 h. Then biofilms were washed with PBS with cautions three times and stained with LIVE/DEAD™ BacLight™ Bacterial Viability Kit (L7012, Invitrogen, United States) according to the manufacturer’s instructions in constant with previous literature (Tawakoli et al., 2013), and the biofilm samples were then imaged by confocal laser scanning microscopy system (CLSM TCS XP8, Leica, Germany).

#### Scanning electron microscopy of biofilm

The 24-h MRSA USA300 biofilm challenged with antimicrobial combination on the cell slides for 24 h was rinsed 3 times with PBS. Then fixed with 3% glutaraldehyde for 5 h at 4°C, and then gently rinsed with saline 3 times for 15 min each. The 12-well cell slides were dehydrated twice for 5 min with each gradient of 30%, 50%, 70%, 90%, 95%, and 100% ethanol, respectively. Ethanol was replaced with isoamyl acetate 2 times for 5 min each. The samples were dried using a critical point drier (HCP-2, Hitachi, Japan). The samples were plated on a copper table with an automatic gold plating apparatus, and the bacterial morphology was observed by an SEM (Regulus 8100, Hitachi, Japan).

### *In vivo* study of antimicrobial and anti-inflammatory effect

#### Animal model and cocktail treatment

Animal experiments were carried out in accordance with the principles and guidelines of the Animal Ethics Committee of the Guangzhou Huateng Education Incorporation and was acknowledged by the First Affiliated Hospital of Sun Yet-sen University. 48 eight-week-old male C57BL/6 J mice (wild type) used in this experiment were purchased from the Experimental Animal Center of the First Hospital of Sun Yat-sen University (Guangzhou, China) and weighed ~22.05 ± 1.87 g. All animals were housed in individual ventilated cages under controlled conditions (temperature 22°C–24°C, relative humidity 40%–70%), with free access to food and water and 12-h circadian rhythm.

**Figure 1:**
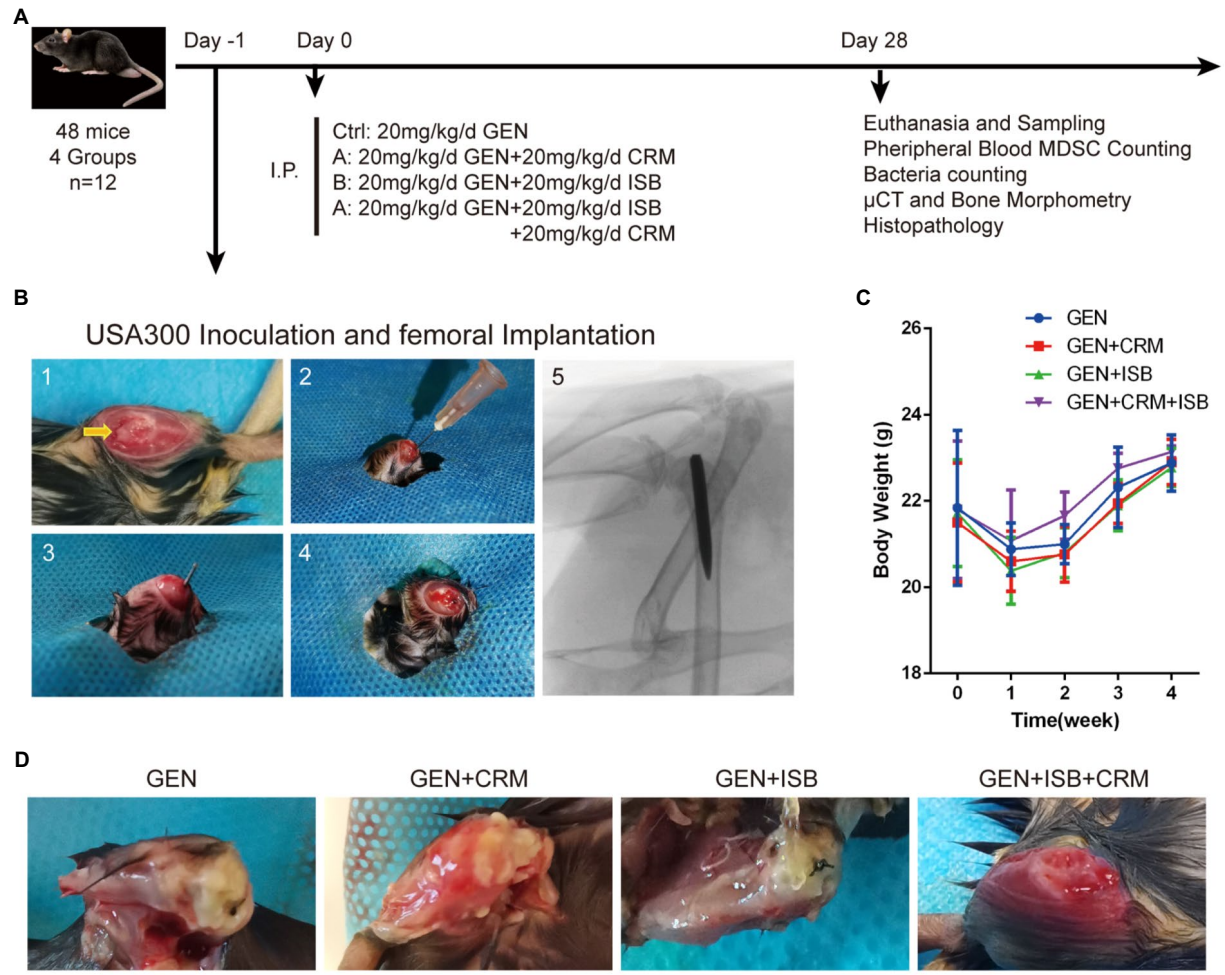
Orthopedic implant-related infection animal model of cocktail of isobavachalcone and curcumin with gentamycin against MRSA. **(A)** *In vivo* study design. **(B)** Surgical procedures of distal femoral implant-related infection mouse model. 1–5 refer to: (1) Distal femoral implant using a trans-knee approach, after incise internally alongside patella and externally dislocating patella to expose the distal femoral articular surface, and the entry point, intercondylar notch, was shown by the yellow arrow. (2) 25-gauge needle was used to open a corridor into the femoral medullary canal. (3) The customized 0.6 mm diameter needle was implanted after the inoculation of 5 μl MRSA USA300 inoculum suspension. (4) The caudal end of the implant was left about 1 mm outside the femur after implantation. (5) The representative X-ray image of implant well positioned in the medullary canal and distal femur. **(C)** Body weight of animals throughout *in vivo* experiment. **(D)** Representative appearance of distal femurs of mice 28 days after intraperitoneal administration of different groups of antimicrobials combinations, gentamicin served as control. I.P., intraperitoneal injection; GEN, Gentamycin; ISB, Isobavachalcone; CRM, Curcumin.

Animals were randomized and divided into 4 equal groups according to post-surgery treatments, including a control group (MRSA-infected + 20 mg/kg/day Gentamicin) and 3 different treatment groups (*n* = 12 per group), including: (A) MRSA-infected + 20 mg/kg/day Gentamicin (GEN) + 20 mg/kg/day Curcumin (CRM); (B) MRSA-infected + 20 mg/kg/day GEN + 20 mg/kg/day Isobavachalcone (ISB); (D) MRSA-infected + 20 mg/kg/day GEN + 20 mg/kg/day CRM + 20 mg/kg/day ISB (Figure 1A). According to *in vitro* results, USA300 was used as pathogen in mouse model, which is one of the most clinically-related MRSA strains in biofilm research, especially in ODRI (Lauderdale et al., 2010). As the pharmacokinetics of ISB and CRM in bone and bone marrow tissue is not clear, no previous report reveals the local drug concentrations in bone tissue and bone marrow after subcutaneous administration. The dosage of ISB (Wang X. et al., 2021) and CRM (Wang et al., 2016; Liu et al., 2019) in mouse model were chosen according to previous literature in order to achieve either anti-bacterial, anti-inflammatory, or anti-osteoporosis, respectively. The Dosage of gentamicin in the animal experiment was chosen according to previous *in vivo* study (Espersen et al., 1994), and is much lower than that can induce acute kidney injury (Huang et al., 2020).

The distal femoral implant was inserted using a trans-knee approach established in intramedullary nailing mice model (as in Figure 1B) and widely used in the ODRI mouse model (Bernthal et al., 2010; Lovati et al., 2013). Animals were fasted with free access to water for 8 h prior to surgery. Animals were anesthetized under pentobarbital sodium (80 mg/kg, i.p.). After disinfection with 75% alcohol and povidone iodine, a medial parapatellar arthrotomy was used to approach the intercondylar notch, and a 25 gauge syringe needle (Ø 0.5 mm) was used as drill to breakthrough subchondral bone, a 27 gauge insulin syringe needle was carefully inserted into for 3–4 mm without expansion to open a retrograde corridor into the femoral medullary canal. When there is no continuous bleeding, switch to a Hamilton microinjector with a 30G needle (25 μl) to slowly inject 5 μl of MRSA USA300 suspension (1–2 × 10^6^ CFU/ml, prepared as aforementioned) into the medullary canal to achieve a total bacterial inoculum of 0.5–1 × 10^4^ CFU per animal. A customized Ø 0.6 mm, 7–8 mm long Kirschner needle was then implanted retrogradely into the femoral medullary canal through the corridor with 1 mm protruded into the joint space. The patella was then repositioned and incision was closed layer by layer with 4–0 synthetic sutures.

Animals were housed in original environment after awakening from anesthesia. One day after surgery and MRSA USA300 inoculation, antibiotics and ISB, CRM, or their combination were intraperitoneally administrated for 4 weeks according to the study design, followed by a daily subcutaneous administration of Meloxicam for 5 days post-surgery. All animals were monitored daily for general status and welfare, and checked weekly to record data on body weight, signs of wound healing, swelling, infection, pain, and suffering. After 4 weeks, the mice were euthanized to perform further investigations.

#### MDSCs in blood analysis

To determine the MDSCs population in the peripheral blood of mice, 1 ml of peripheral blood samples were collected using cardiac puncture from 6 animals per group at the end of animal experiment for flow cytometry analysis. After lysis of erythrocytes using erythrocyte lysis solution, the remaining leukocytes were resuspended in PBS containing 2% FBS and then incubated for 30 min at 4°C with rat anti-mouse CD11b-FITC (ab24874, Abcam, United States) and rat anti-mouse Gr-1-PE (12-9668-82, Thermo Fisher, United States) at the manufacturer’s recommended concentration. MDSCs in mouse peripheral blood was analyzed by co-staining with rat anti-mouse CD11b-FITC (ab24874, Abcam, United States), Gr-1-PE (12-9668-82, Thermo Fisher, United States). BD FACSCantoII flow cytometry data were further analyzed using FACSDiva software (BD Biosciences). The number of cells analyzed per sample was ~10,000. Analysis was performed using FlowJo software (Tree Star, United States).

#### μCT analysis

Distal femur specimens were collected and fixed in 4% paraformaldehyde. The bone microarchitectures surrounding implant of the distal femurs were analyzed by a desktop μCT SkyScan1276 (Bruker Micro CT, Belgium). In the present study, μCT scanner was operated at 85 kV and 200 μA, with a scaled image pixel size of 10.0 μm, 1 mm Al filter was used for optimal image contrast, and spiral scanning was used to reduce metal artifacts. Images were reconstructed and processed with Software Nrecon (Bruker micro-CT, Belgium). Software CTAn (Bruker micro-CT, Belgium) was used to perform bone morphometry analysis. Trabecular bone was separated from cortical bone by free-drawing region of interest (ROI), and metallic implant in the distal femur was ruled out by setting bony binary selection threshold as 75–150. Volume of interest (VOI, 2 mm proximal to the distal metaphyseal line) was chosen within 200 continuous slices. We performed bone morphologic measurements in CTAn and obtained corresponding parameters, including trabecular bone volume fraction (BV/TV; %), trabecular thickness (Tb.Th; mm), trabecular number (Tb.N; mm^−1^), bone surface density (BS/TV; mm^−1^), trabecular separation (Tb.Sp; mm), and bone mineral density (BMD; mg/cm^3^). Then, the 3D models of VOI were reconstructed with CTAn and then visualized in CTVol (Bruker micro-CT, Belgium). The operators conducting the μCT analysis were blinded to the treatments associated with samples.

#### Histological assessment and immunofluorescence staining

After μCT scanning, 6 femur specimens per group were fixed in 4% paraformaldehyde for at least 48 h, and washed three times with PBS buffer. Samples were decalcified in 10% EDTA (E1171, Solarbio, China) on a shaker at 4°C, the solution was changed every 3 days for 21 days. The specimens were then washed 3 times with PBS buffer and subjected to dehydrated, paraffin-embedded, and sectioned 4 μm slides. HE staining was performed in order to analyze the inflammation and general changes of bone microstructure at histological and cytological levels.

Immunofluorescence staining was applied to analyze MDSCs polarization to M1 according to standard protocols. The sections were incubated at 4°C overnight with primary antibodies rabbit anti-mouse CD11b (ab184308, 1:500, Abcam, United States) and rabbit anti-mouse CXCL10 (10H11L3, 1:500, Thermo Fisher, United States) for multiplex, respectively; the corresponding secondary antibodies were added onto the sections for 1 h. For immunofluorescence, slides were counterstained with DAPI. The slide images were observed and captured by Eclipse Ti-SR microscope (Nikon, Japan). ImageJ was used for the quantitative analysis if necessary.

#### Quantification of bacteria in bone

Surgical dissection of femur for bacteria counting was performed under sterile conditions at 4°C. Femur specimens were weighed and cut into small pieces under aseptic conditions using small Petri dishes, then pulverized using a tissue grinder (DHS TL2010S, China). Tissue samples were resuspended with D/E neutralizing broth and then sonicated for 15 min along with the implanted Kirschner pins to detach residual bacterial biofilm from both bone tissue and implant. The sonicated suspension was immediately inoculated onto TSA agar plates by gradient dilution and incubated at 37°C for 24 h. Colony counting was performed to quantify the amount of bacteria per unit mass of femur (CFU/mg).

### Statistical analysis

Statistical analysis was performed by using the GraphPad Prism 6 (GraphPad Software, CA, United States). All data were presented as mean ± SD. All error bars in figures represent SD Group comparison was made by using unpaired, two-tailed Student’s *t*-test. For all statistical analyses, ^∗^*p* < 0.05 was considered to be significant.

## Results

### Synergy of ISB or CRM with antibiotics against planktonic *Staphylococcus aureus*

**Figure 2:**
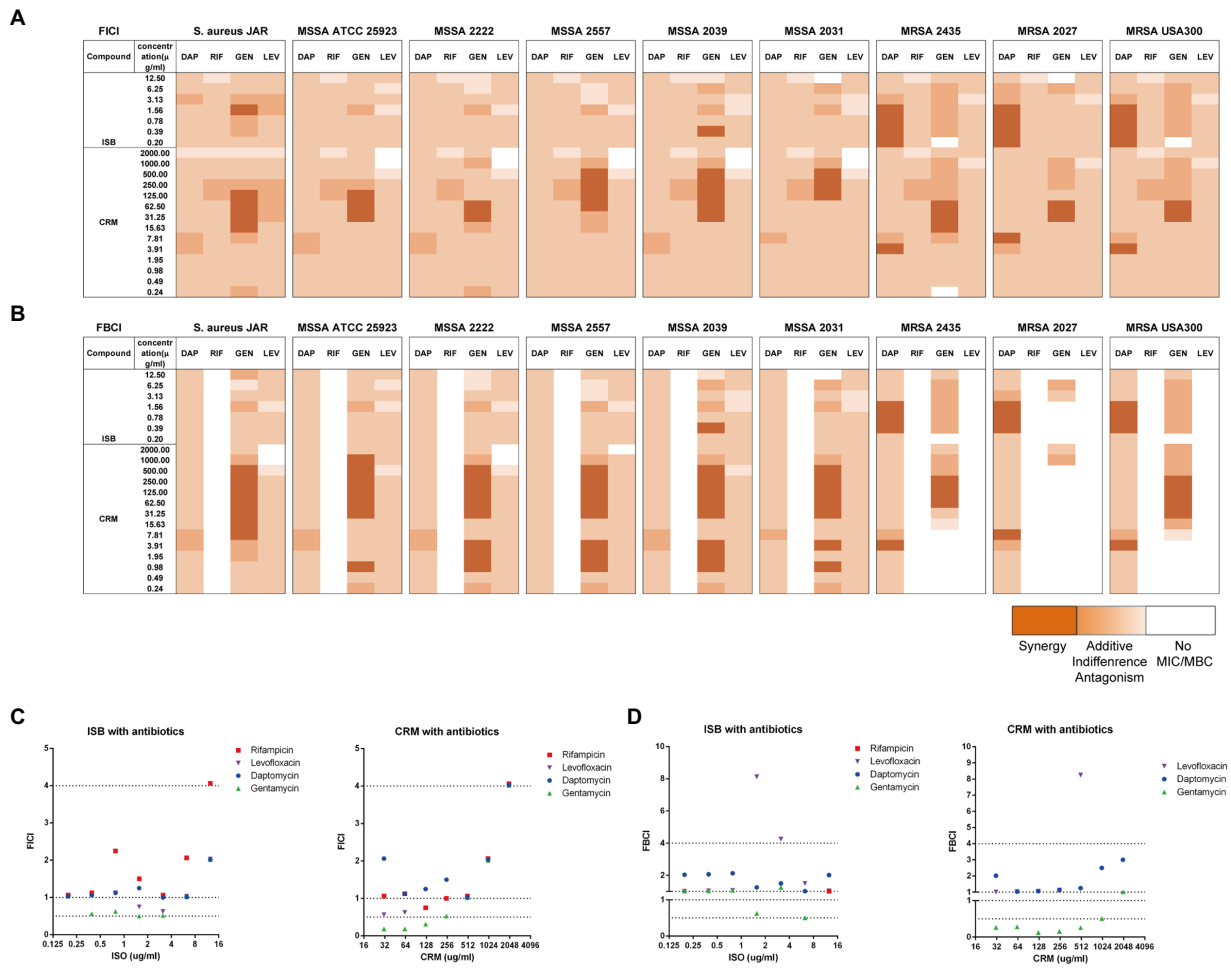
Interaction of Isobavachalcone and Curcumin with Antibiotics on MSSA and MRSA strains. **(A,B)** Heatmaps of fractional inhibitory concentration index (FICI) and fractional bactericidal concentration index (FBCI) of combination of gentamycin with isobavachalcone or curcumin, darker orange color indicates interaction is superimposed or synergistic. **(C,D)** FICI and FBCI of Isobavachalcone and Curcumin with antibiotics against *Staphylococcus aureus* JAR strain, respectively. FICI ≤ 0.5 represents synergy, FICI > 0.5–4 represents no synergistic interaction, and FICI > 4.0 represents  antagonism. FICI: fractional inhibition concentration index; FBCI: fractional bactericidal concentration index; DAP: Daptomycin with 50 μg/ml Ca2+; RIF: Rifampicin; GEN: Gentamycin; LEV: Levofloxacin; ISB: Isobavachalcone; CRM: Curcumin; MSSA: Methicillin-susceptible *Staphylococcus aureus*; MRSA: Methicillin-resistant *Staphylococcus aureus*.

Prior to biofilm challenge assays, it is critical to verify the interactions between ISB or CRM and antimicrobials against planktonic *S. aureus*. Daptomycin (DAP), rifampicin (RIF), gentamicin (GEN), and levofloxacin (LEV) were chosen as antimicrobials according to our preliminary biofilm challenge data on the *in vitro* 24 h model, and experimental concentrations were chosen according to their MICs and MBCs (Supplementary data). The fractional inhibition concentration index (FICI), as described in the methodology, reflects the combined inhibitory effect of the two antimicrobial substances. The FICIs of Isobavachalcone (ISB) or Curcumin (CRM) with four antibiotics were calculated (Figure 2A), and the results implied that in all combinations with four antibiotics the MICs were acquired within the experimental concentration range. There is synergy (FICI ≤ 0.5) in combinations of GEN with ISB (0.39, 1.56 μg/ml), GEN with CRM (15.6–500 μg/ml) against six MSSAs. For MRSAs, all combinations with antibiotics also had the MIC cut-off values, and synergy existed only in the combinations of DAP with ISB (0.2–1.56 μg/ml) and DAP with CRM (3.9, 7.8 μg/ml); ISB and CRM were synergistic only in the combination of GEN with CRM (15.6–62.5 μg/ml). In the case of a well-investigated clinical isolated MSSA strain *S. aureus* JAR (Campoccia et al., 2008; Figure 2C), the combinations with synergy are GEN with CRM (15.6, 32–128 μg/ml), GEN and ISB (1.56 μg/ml).

The fractional bactericidal concentration index (FBCI) reflects the combined bactericidal effect of the two antimicrobial substances. The FBCIs of ISB or CRM with antibiotics were also calculated (Figure 2B), which showed that the combinations with all four antibiotics, except RIF could acquire MBCs for all MSSAs tested within the experimental concentration range. The combination of GEN with CRM (0.98–3.9, 7.98–500 μg/ml) are synergistic (FBCI ≤ 0.5). The absence of bactericidal effect of RIF-containing combinations against all MSSA and MRSA may be due to the low concentration range (0.001–0.5 μg/ml) of RIF, which is concentration-dependent (Hirai et al., 2016) and required a higher concentration (>0.5 μg/ml for MSSA and >128 μg/ml for MRSA). For MRSAs, all combinations with DAP acquire MBC cut-off values and there is synergy in the combinations of DAP with ISB (0.39–1.56 μg/ml) and DAP with CRM (3.9, 7.8 μg/ml). GEN-containing combinations with ISB and CRM against MRSAs reach the MBC cut-off values, and the combination of GEN with CRM (62.5–250 μg/ml) is synergistic. The low concentration of GEN (0.16–8 μg/ml) tested may be the reason for the absence of bactericidal effect on three MRSA strains (Atashbeyk et al., 2014). Neither the combination of RIF nor LEV eradicate three MRSA strains, in addition to the low concentration of RIF as previously explained, concentrations of LEV in this study (0.079–4 μg/ml) is lower than MBC (8–20 μg/ml) against MRSA (Drago et al., 2001; John et al., 2009). In the case of *S. aureus* JAR strain (Figure 2D), the only combination with significant synergy is GEN with CRM (7.8–500 μg/ml). In summary, FICIs and FBCIs indicate the combination of GEN with ISB or CRM is worthy of further investigation.

### Synergy of ISB or CRM with antibiotics against MSSA biofilm

**Figure 3:**
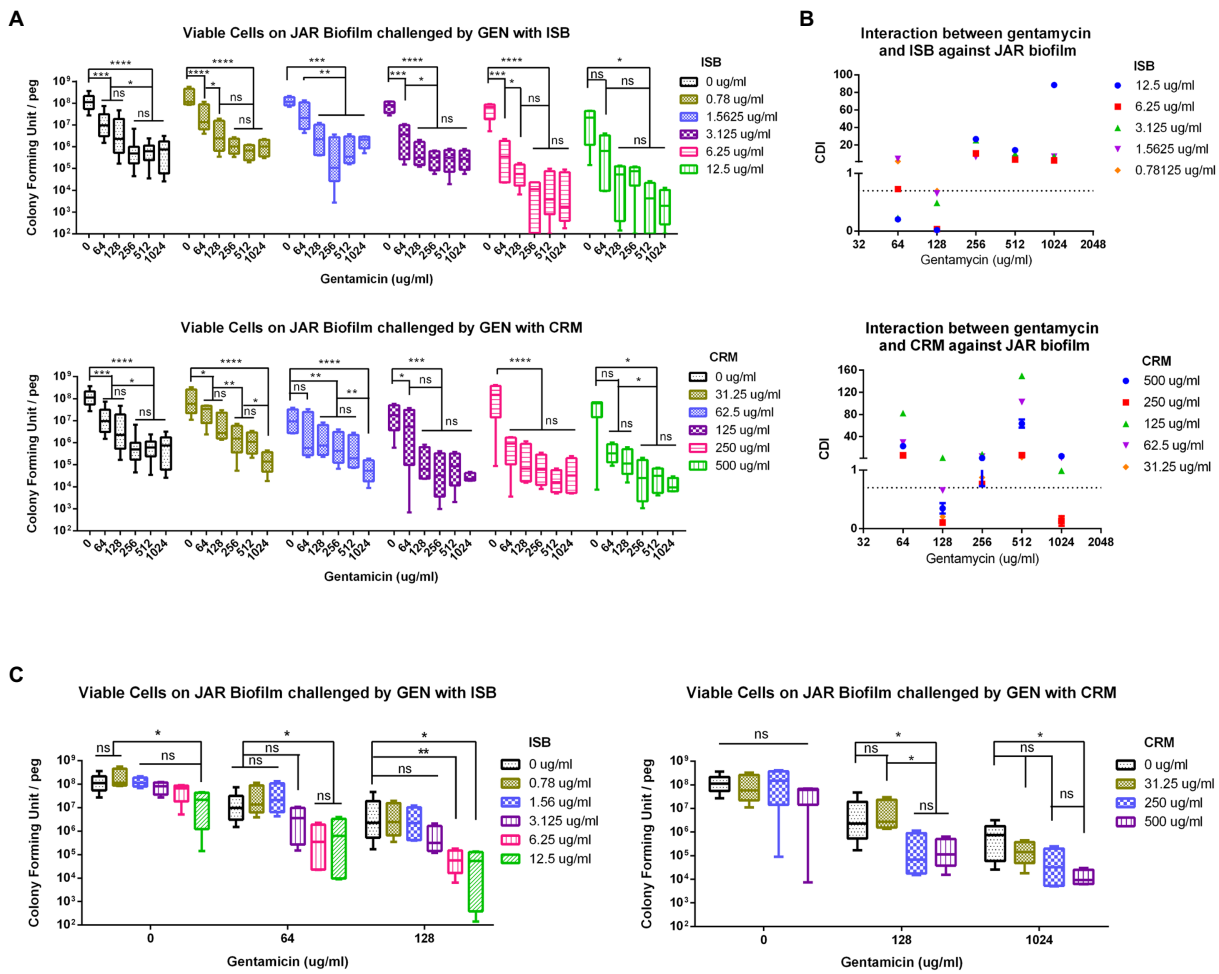
Interaction of isobavachalcone and curcumin with Gentamycin against *Staphylococcus aureus* JAR biofilm. **(A)** Residual *S. aureus* JAR on Biofilm challenged by combination of Gentamycin with Isobavachalcone or Curcumin. **(B)** Coefficient of Drug Interaction (CDI) of the combination of Gentamycin with isobavachalcone or curcumin. **(C)** Synergistic Combination of isobavachalcone with Gentamycin, and Curcumin with Gentamycin. CDI < 1, =1 or >1 indicate synergy, superposition or antagonism, respectively, CDI < 0.7 indicates significant synergism. Data were presented as mean ± SD. ^*^ represents *p* < 0.05, ^**^ represents *p* < 0.01, ^***^ represents *p* < 0.001, ^****^ represents *p* < 0.001, when compared to ISB/CRM with the same Gentamycin concentration, ns represents no significance. GEN, Gentamycin; ISB, Isobavachalcone; CRM, Curcumin; CDI, Coefficient of Drug Interaction.

To further analyze the synergistic efficacy of ISB/CRM and antibiotics against *S. aureus* biofilms, the MBEC™ Assay was performed to quantify the surviving bacteria on MSSA JAR biofilms challenged by the combinations of GEN with CRM or ISB. Viable bacteria in biofilms were quantified after challenge (Figure 3A), and it shows that for all tested concentrations of ISB and CRM, the number of bacteria on biofilm decreased with increasing antibiotics concentration, where the MBEC for gentamicin (GEN) is >1,024 μg/ml, which is consistent with previous study (Kaneko et al., 2020). ISB and CRM alone cannot eradicate biofilm without GEN. As the concentration of CRM and ISB increased, the number of viable bacteria of MSSA JAR biofilm show a decreasing trend (Figure 3A) at identical GEN concentration. Although the mean value of CFU/per failed to decrease to zero, higher ISB/CRM concentration significantly enhance anti-biofilm efficacy of GEN and a lower MBEC of GEN than normal (>1,024 μg/ml) can be observed on some samples, where biofilm was entirely eliminated. Thus GEN + CRM and GEN + ISB may have a synergistic eradication against MSSA JAR biofilm.

In present study, all combinations have no MBEC cut-off value, in order to further analyze the synergistic effect of those combinations aforementioned, the combinations with synergy on *S. aureus* biofilm were identified by Coefficient of Drug Interaction (CDI), commonly used in other pharmacodynamic fields, was introduced as a parameter as described in methods. The results revealed that GEN with CRM or ISB were synergistic (Figure 3B), and with significant synergy (CDI < 0.7) at specific concentrations, namely GEN (128 μg/ml) + CRM (31.25, 62.5, 250, 500 μg/ml), GEN (64,128 μg/ml) + ISB (1.56, 3.13, 6.25, 12.5 μg/ml; Figure 3B). Further analysis of synergistic combinations suggests that in the combination of GEN + ISB, with GEN at 64 and 128 μg/ml, when ISB rises above 6.25 μg/ml, the number of bacteria on biofilm reduced significantly by log-2 to log-3 compared to control (Figure 3C). In the combination of GEN + CRM, the reduction of bacteria in biofilm by CRM alone was also not statistically significant. Whereas to GEN at 128 and 1,024 μg/ml, with CRM concentration increased (≥250 μg/ml with 128 μg/ml of GEN, ≥500 μg/ml with 1,024 μg/ml of GEN), bacteria on biofilm could be significantly reduced up to log-2–log-4 compared to control. There is also a significant reduction in colony counts between GEN at 0, 128, and 1,024 μg/ml with high concentrations of CRM (Figure 3C). Overall, MBEC assays indicate the combination of ISB or CRM is efficient in enhancing the susceptibility of MSSA to Gentamicin.

### Synergy of cocktail of ISB, CRM with gentamicin against MRSA biofilm

**Figure 4:**
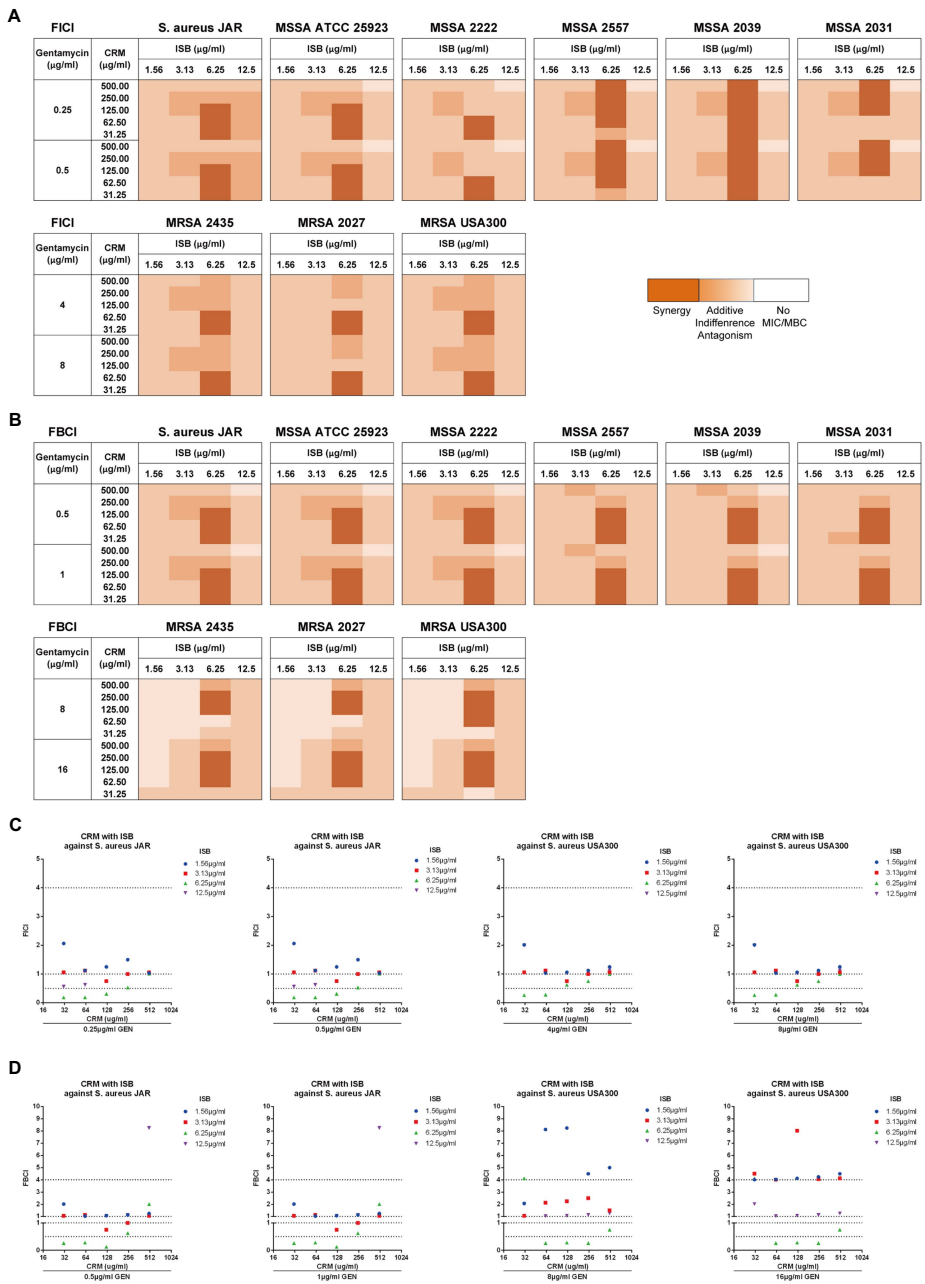
EUCAST checkerboard assay showed isobavachalcone and curcumin interact synergistically with presence of gentamycin against planktonic *Staphylococcus aureus*. **(A,B)** Heatmaps of fractional inhibitory concentration index (FICI) and fractional bactericidal concentration index (FBCI) of combination of isobavachalcone and curcumin, darker orange color indicates interaction is superimposed or synergistic. **(C)** FICI of isobavachalcone with curcumin against *S. aureus* JAR and USA300 strain. **(D)** FBCI of isobavachalcone with curcumin against *S. aureus* JAR and USA300 strain. FICI ≤ 0.5 represents synergy, FICI > 0.5–4 represents no synergistic interaction, and FICI > 4.0 represents antagonism. CDI < 1, =1 or >1 indicate synergy, superposition or antagonism, respectively, CDI < 0.7 indicates significant synergism. Data were presented as mean ± SD. FICI, fractional inhibition concentration index; FBCI, fractional bactericidal concentration index; GEN, Gentamycin; ISB, Isobavachalcone; CRM, Curcumin; CDI, Coefficient of Drug Interaction; MSSA, Methicillin-susceptible *Staphylococcus aureus*; MRSA, Methicillin-resistant *Staphylococcus aureus*.

In order to first analyze the synergistic efficacy of ISB with CRM against planktonic *S. aureus*, the EUCAST checkerboard assay was conducted with presence of Gentamicin range from sub-MIC against MSSA (0.25 μg/ml) to sub-MIC against MRSA (8 μg/ml) for FICI analysis, and from sub-MBC against MSSA (0.5 μg/ml) to sub-MBC against MRSA (16 μg/ml) for FBCI analysis. The FICI analysis shows that synergistic inhibitory effect exists between combinations of 6.25 μg/ml ISB with 31.25–500 μg/ml CRM against various MSSA strains, and with 31.25–62.5 μg/ml CRM against MRSA strains with 64 or 128 μg/ml Gentamicin presence (Figure 4A). The FBICs also show similar synergy pattern as FICI, and synergy exists between combinations of 6.25 μg/ml ISB with 31.25–125 μg/ml CRM against various MSSA strains, and with 62.5–250 μg/ml CRM against MRSA strains (Figure 4B). Not all synergistic combinations are with the same concentration range of CRM, which is bacteria strain-dependent against MSSAs; however, the synergistic concentration range for CRM is relatively more consistent for different MRSAs. As the typical clinical isolated prolific biofilm former MSSA JAR strain (Campoccia et al., 2008), synergistic combinations are 6.25 μg/ml ISB with 31.5–125 μg/ml CRM with presence of Gentamicin. Another standard prolific biofilm former MRSA USA300 (Vanhommerig et al., 2014), 6.25 μg/ml ISB with 31.5–62.5 μg/ml CRM exhibits synergistic inhibition with gentamicin, while bactericidal synergy requires 6.25 μg/ml ISB with 62.5–250 μg/ml CRM with presence of Gentamicin (Figures 4C,D). Notably, In the present study, combinations of ISB and CRM with other concentrations of Gentamicin did not show synergy both in inhibition and bactericidal effect (data not showed), which may be related to the insufficient antibacterial potency of lower concentrations (<sub-MIC/sub-MBC) of gentamicin (data not showed), while in this study higher concentrations of gentamicin (≥MIC/MBC) had independent antibacterial effects against planktonic *S. aureus*, and the synergistic effects of ISB and CRM were difficult to manifest, therefore, combinations with gentamicin higher than MIC and MBC were not investigated. Given the synergy between ISB with 64, 128 μg/ml GEN, and CRM with 128, 1,024 μg/ml GEN against MSSA biofilm (Figure 3B), and the significant reduction of bacteria on biofilm treated with combination of ISB/CRM with 128 μg/ml GEN revealed in previous experiments (Figure 3C), thus we tested the combination of ISB + CRM with a 128 μg/ml GEN concentration in biofilm experiment.

**Figure 5:**
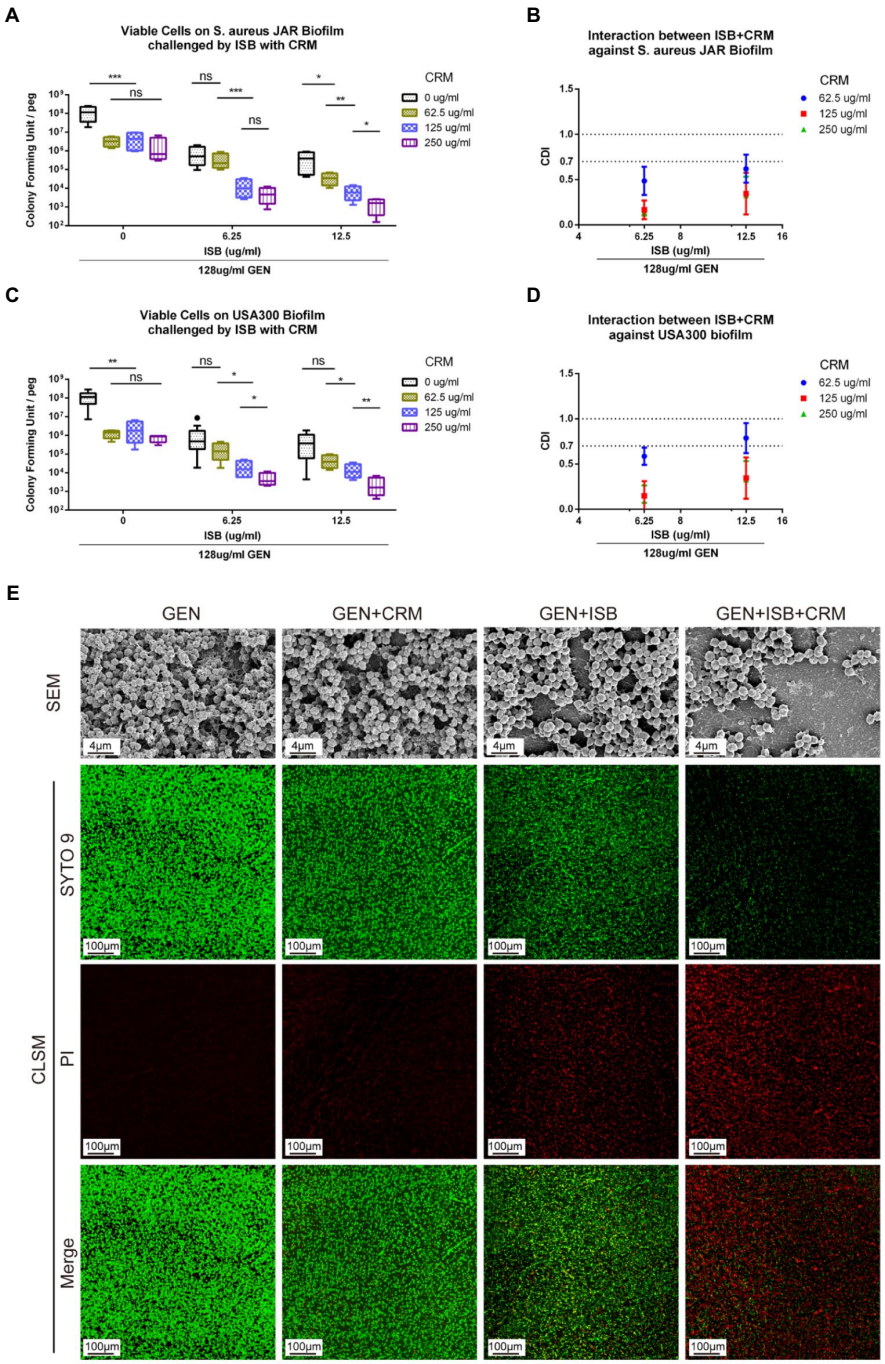
*In vitro* Eradication of *Staphylococcus aureus* biofilms by the GEN-ISB-CRM combination. **(A,C)** Quantification of residual viable cells on *S. aureus* JAR biofilm and USA300 biofilm, respectively. **(B,D)** Coefficient of Drug Interaction (CDI) shows synergy between Isobavachalcone and Curcumin with 128 μg/ml Gentamycin against *S. aureus* JAR biofilm and USA300 biofilm, respectively. **(E)** Representative characteristics of MRSA USA300 biofilm after 24 h challenge. SEM: biofilms cultured at 37°C for 24 h were treated with 128 μg/ml GEN (control), 128 μg/ml GEN + 125 μg/ml CRM, 128 μg/ml GEN + 6.25 μg/ml ISB, 128 μg/ml GEN + 125 μg/ml CRM + 6.25 μg/ml ISB for 24 h, respectively. All SEM images were captured at EHT = 10 kV, Mag = 5KX. CLSM: representative confocal laser scanning microscopy (100× oil immersion) images of LIVE/DEAD^®^ BacLight staining showing the synergistic effect of different drug combinations on USA300 biofilm challenged for 24 h, green: live cells; red: dead cells. Data were presented as mean ± SD. ^*^ represents *p* < 0.05, ^**^ represents *p* < 0.01, ^***^ represents *p* < 0.01 when compared to 0 μg/ml CRM with the same ISB concentration group, ns represents no significance. GEN, Gentamycin; ISB, Isobavachalcone; CRM, Curcumin; CDI, Coefficient of Drug Interaction; SEM, Scanning Electron Microscopy; CLSM, Confocal Laser Scanning Microscopy.

To investigate whether there is synergy of ISB, CRM, and Gentamicin against *S. aureus* JAR and MRSA USA300 biofilm, the MBEC™ assays of their combinations were performed with presence of Gentamicin at 128 μg/ml, which is proven to be synergistic with ISB/CRM against MSSA biofilm as aforementioned, respectively. Quantification of viable cells both on 24 h *S. aureus* JAR or USA300 biofilm challenged by the cocktails reveals a significant decreased MRSA with increasing CRM concentration with both 6.25 and 12.5 μg/ml ISB, and with Gentamicin at 128 μg/ml, whereas although ISB exhibit significant enhancement of susceptibility of USA300 biofilm to 128 μg/ml Gentamicin and CRM (62.5–250 μg/ml), this enhanced eradication effect was not in a ISB concentration-dependent manner (Figures 5A,C). Further analysis of Coefficient of Drug Interaction (CDI) showed that cocktails of 6.25–12.5 μg/ml ISB and 62.5–250 μg/ml CRM with 128 μg/ml Gentamicin are all synergistic against both 24 h *S. aureus* JAR and MRSA USA300 biofilm (Figures 5B,D). Overall, *S. aureus* JAR biofilm treated with the same formulae contains less viable bacteria than USA300 biofilm, especially with 6.25 μg/ml ISB and 125–250 μg/ml CRM (Figures 5A,C); however, their CDIs were very similar. The synergistic eradication of 24 h MRSA USA300 biofilm was also confirmed through SEM and live/dead cell staining (Figure 5E), where cocktail of 128 μg/ml Gentamicin together with 125 μg/ml CRM + 6.25 μg/ml ISB showed promising biofilm eradication effect. We also tested the ISB + CRM combination with 64 and 1,024 μg/ml GEN, which was proven to be synergistic with ISB or CRM against MSSA biofilm. However, there is neither significant reduction of bacteria on biofilm (probably due to lower 64 μg/ml GEN) nor synergy between the three antimicrobials (1,024 μg/ml GEN; data not showed). The results also indicates the biofilm eradication effect of Gentamicin with ISB is more potent than Gentamicin w/o CRM, which is consistent with previous MSSA biofilm experiments.

### Cocktail therapy of ISB and CRM reduce Osteolysis during device-related infection

After surgery, all the animals had normal feeding during the *in vivo* experiment; however, as femoral intramedullary implant infection progressed, mice in Gentamicin and Gentamicin + CRM groups gradually exhibited restricted knee joint mobility of the affected hind limb and line to reduce movement with affected limb. All mice except for those in Gentamicin + ISB + CRM group showed various degrees of swelling and pain in the knee joint of the limb with implant. We monitored the body weight of mice in each group before operation and 1–4 week postoperation (Figure 1C). There was no significant difference in the initial body weight of the mice between all four groups of experimental animals. Generally, the body weight of mice in all groups decreased significantly (*p* < 0.05) 1 week post-surgery due to surgical trauma, and then with rehabilitation, the body weight of mice gradually increased until they were euthanized. Although body weight of mice in Gentamicin group was expected to be gradually lower than other three groups as MRSA infection progressed, there was no significant difference in body weight among each groups at each time point, suggesting that the dosage of gentamicin used in the present study may be sufficient to stop the progression of topical infection to the systemic level in an animal model with a relatively strong resistance to infection, like murines. After 28 days of antimicrobials administration, when femur with implant was sampled after sacrifice, significant soft tissue abscess penetration into the subcutaneous tissue was observed in the Gentamicin and Gentamicin+CRM groups, accompanied by significant soft tissue congestion and edema around the knee joint, while no significant local soft tissue abscess formation was observed in the GEN + ISB group, but with mild soft tissue thickening and edema. Soft tissues around knee joint of mice in Gentamicin+ISB + CRM group were only mildly congested and clearly hierarchical without edema in appearance (Figure 1D). No obvious hepatorenal toxicity of cocktail therapy compared to gentamicin alone was observed (serum biochemical analysis data not showed).

**Figure 6:**
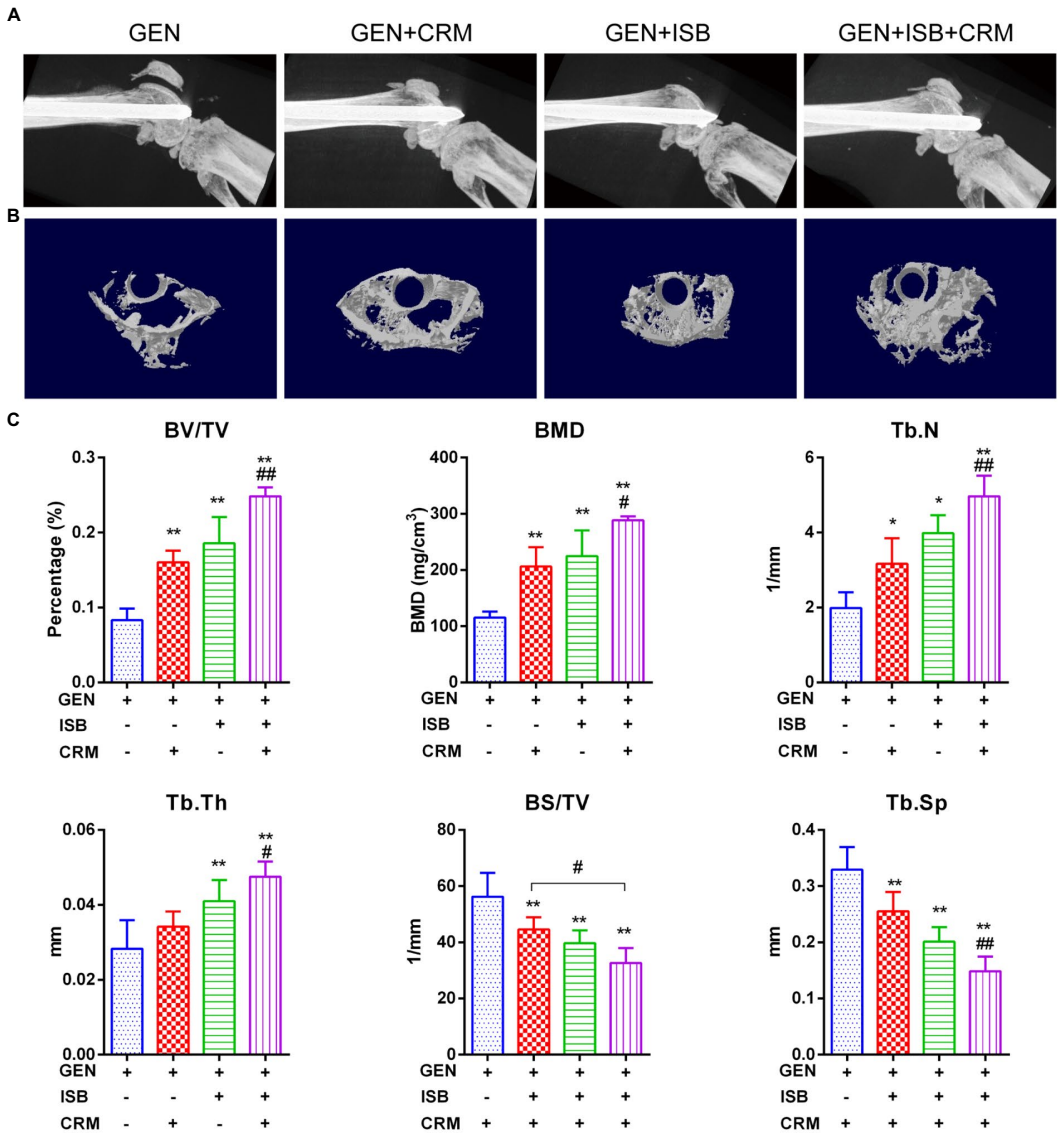
Micro Computed tomography (μCT) imaging suggests protection of cocktail therapy against osteolysis in orthopedic implant-related infections due to MRSA. **(A)** Representative X-ray images of distal femur of mouse with intramedullary implant. **(B)** Representative images of trans axial 3D reconstructed micro-architecture of distal femur around implant. **(C)** Bone morphometry in distal femur shows protection of combination of Isobavachalcone and Curcumin against osteolysis during orthopedic implant-related infection, related parameters including percent bone volume (BV/TV), trabecular thickness (Tb.Th), trabecular number (Tb.N), and trabecular separation (Tb.Sp), bone mineral density (BMD) and bone surface density (BS/TV) were measured, data were presented as mean ± SD, ^*^ represents *p* < 0.05 and ^**^ represents *p* < 0.01 when compared to Gentamicin group; # represents *p* < 0.05 and ## represents *p* < 0.01 when GEN + ISB + CRM group compared to GEN + ISB/CRM group; GEN, 20 mg/kg/day Gentamycin; ISB, 20 mg/kg/day Isobavachalcone; CRM, 20 mg/kg/day Curcumin.

μCT imaging and bone morphometry of distal femur suggests the protection of cocktail therapy against osteolysis in orthopedic device-related infections due to MRSA (Figure 6). X-ray imaging showed mild to moderate bone absorption both in Gentamicin + CRM and Gentamicin + ISB group, and moderate to severe absorption in Gentamicin group accompanied with commonly seen periosteal reaction and in some cases with fracture (Figure 6A). 3D reconstructed micro-architecture of distal femoral bone around implant indicates osteolysis and microstructural destruction in the femur of infected mice (Figure 6B), which was also confirmed by quantitative bone morphometry. BV/TV, BMD, Tb.N, and Tb.Th were significantly lower (*p* < 0.05) in the Gentamicin-alone treated mice, while BS/TV and Tb.Sp were higher (Figure 6C). Treatment with Gentamicin with CRM/ISB significantly increased morphometry parameters indicating trabecular bone microstructure, while cocktail therapy with Gentamicin+ISB + CRM can further enhance such protection significantly.

### Cocktail therapy enhance reduction of MDSC M1 polarization and eradication of MRSA biofilm *in vivo*

**Figure 7:**
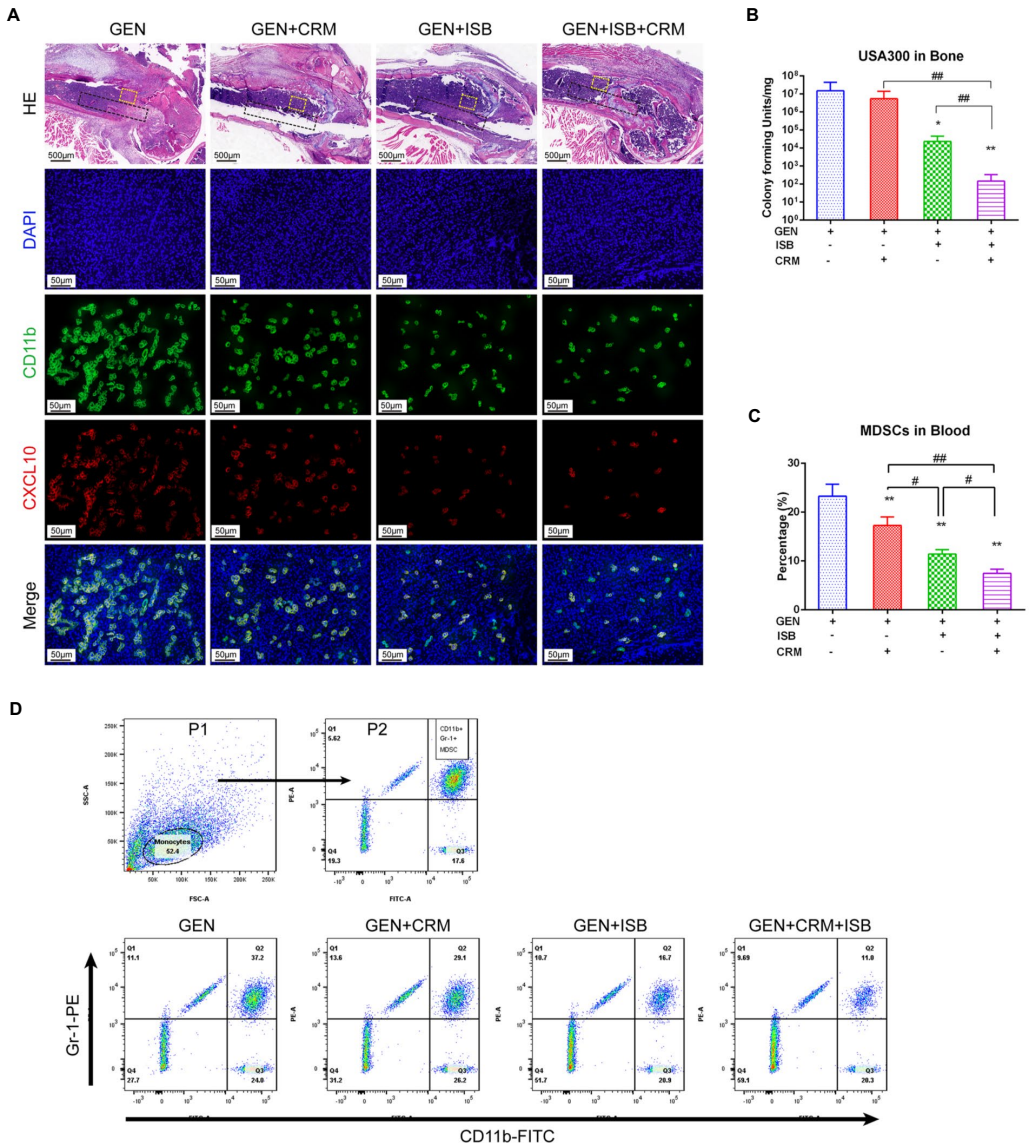
Cocktail therapy of Gentamicin with Isobavachalcone and Curcumin alleviate local tissue inflammation, enhance reduction of MDSC M1 Polarization and Eradication of MRSA biofilm *in vivo*. **(A)** HE and immunofluorescent staining of distal femur suggest reduction of tissue inflammation and M1 polarization of MDSC, green represents CD11b, a key marker to identify MDSC, while red represents CXCL10, a key marker of activated M1, he black dashed box indicates the approximate location of the implant, and the yellow dashed box range is the area where M1-polarized MDSC in the adjacent implant tissue is observed. **(B)** Quantification of remaining MRSA in bone with implant. **(C)** Flow cytometry analysis showed reduction of MDSC in peripheral blood by Gentamicin with ISB or CRM and cocktail therapy. **(D)** Gating strategy for flow cytometry analysis and representative analysis of frequency of MDSC in peripheral blood. All data were presented as mean ± SD, ^*^ represents *p* < 0.05 and ^**^ represents *p* < 0.01 when compared to Gentamicin group; # represents *p* < 0.05 and ## represents *p* < 0.01 when GEN + ISB + CRM group compared to GEN + ISB/CRM group; GEN, 20 mg/kg/day Gentamycin; ISB, 20 mg/kg/day Isobavachalcone; CRM, 20 mg/kg/day Curcumin.

HE and immunofluorescent staining was used to further verify the alleviation of local tissue inflammation and bacteria in distal femur were quantified to reveal the eradication of MRSA biofilm *in vivo.* H and E staining of distal femur revealed that, except for the Gentamicin+ISB + CRM group, bones around implants infected by MRSA exhibited severe resorption, destruction of the epiphyseal plate, with a large quantity of mononuclear cells filling the medullary canal and bone marrow cavity in between trabecular bones and marked thickening of the soft tissue with leukocyte infiltration (Figure 7A). Osteolysis was significantly reduced after combined treatment with CRM/ISB and gentamicin, compared to the group treated with gentamicin alone, which were consistent with μCT analysis and X-ray findings. In addition, bacterial quantification suggested that the viable MRSA USA300 on the peri-implant bone tissue and implant biofilm decreased significantly after treatment with Gentamicin+ISB compared to Gentamicin alone (*p* < 0.05), and that the cocktail therapy significantly enhance the eradication of MRSA *in vivo* (Figure 7B). In addition, to confirm whether cocktail therapy could inhibit MDSC amplification in peripheral blood in an *in vivo* model of MRSA device-related infection, we quantified the MDSCs in peripheral blood of mice. MDSC level was higher after Gentamicin treatment of MRSA infection, and MDSC amplification in peripheral blood was slightly suppressed after Gentamicin + CRM treatment, but the difference was not statistically significant. The combination of gentamicin and ISB significantly affected MDSC amplification in peripheral blood compared with treatment with gentamicin alone, whereas the cocktail of gentamicin + ISB + CRM significantly reduced the number of MDSC in peripheral blood in mice with MRSA infections (Figures 7C,D). Together, these results suggest that ISB + CRM significantly inhibits MRSA-induced MDSC amplification in orthopedic device-related infections *in vivo*, the combination of ISB + CRM may act as an adjuvant with gentamicin, which administrated alone is ineffective against MRSA biofilms at conventional concentrations, to significantly enhance its antimicrobial effect.

## Discussion

Despite the discovery of penicillin in 1928 and the development of antibiotics, antimicrobial therapy has brought more satisfactory results for patients, but in the last decades we have had to face the emergence of drug-resistant pathogens and the current conventional antibiotic therapy is becoming ineffective for patients (Li and Knetsch, 2018; Xu et al., 2018). *Staphylococcus aureus* is a major cause of orthopedic device-related infections (ODRI) that lead to implant failure, loosening, and reoperation of implants after surgery. In particular, antimicrobial-resistant *S. aureus* (such as Methicillin-resistant *Staphylococcus aureus*, MRSA) is the most difficult to resolve in the clinic (Biedenbach et al., 2004), which are becoming a global public health challenge. Although more advanced antibiotics could be applied in patients, they still have disadvantages such as insusceptibility to resistant strains, increased toxicity at high doses, and long duration (Lebeaux et al., 2014). Therefore, there is an urgent need for research into new therapeutic approaches for drug-resistant pathogens and associated infectious diseases (Microbiology by Numbers, 2011). Bacterial biofilms on the surface of orthopedic implants are considered to be a major cause of antimicrobial resistance because antibodies and bactericidal agents have difficulty penetrating biofilm due to EPS, resulting in a persistent chronic inflammatory response (Flemming et al., 2007; Bjarnsholt et al., 2018), then followed by tissue structural destruction and impaired function. Therefore, there is an urgent need for the development of new drugs, new drug combinations, and alternative therapeutic approaches.

In this study, a standard “biofilm inoculator” (MBEC™ biofilm inoculator) was used *in vitro* to mimic the formation of biofilm by bacteria in a fluid environment on the surface of metal, in this case, bacterial biofilms were generated on the plastic peg on the cover. Previous *in vitro* studies reported no significant differences in the adhesion of *S. aureus*, *S. epidermidis*, and *P. aeruginosa* to the different surfaces of cobalt-chromium meta, ceramic, highly cross-linked polyethylene, and titanium porous-coated acetabular components of commonly used orthopedic prostheses or implants (Slullitel et al., 2018). In our study, four clinically used representative antibiotics with different antimicrobial mechanisms had been initially tested for interaction with isobavachalcone (ISB) and curcumin (CRM), and we found both ISB and CRM exhibit synergy or addition with gentamicin. We further investigate their interaction on six MSSA and three MRSA strains including clinical isolates, and gentamicin was found to be synergistic against planktonic MSSA with curcumin, and is additive against MSSA with isobavachalcone at specific concentrations. Although the daptomycin (DAP) is a much more potent antibiotics against MRSA than gentamicin, and is seemed to be synergistic with isobavachalcone (ISB) against planktonic MSSA, we did not observe any synergy between DAP with ISB against MSSA or MRSA biofilm (data not showed) as for reducing CFU in the biofilm. Gentamicin with curcumin together with isobavachalcone also exhibits synergistic effect in this 24 h formation and 24 h antimicrobials challenge MSSA biofilm model. Therefore, we chose gentamicin for further combinational investigation on the MRSA strains and found that cocktail of 128 μg/ml gentamicin together with 125 μg/ml curcumin + 6.25 μg/ml isobavachalcone showed potent eradication effect with synergy of curcumin and isobavachalcone against USA300 biofilm.

In this “cocktail” of antimicrobials, gentamicin is one of aminoglycosides that has been widely used in ODRI because of its antimicrobial efficacy against non-resistant Staphylococcus, relatively low prevalence of toxicity, and thermal stability. However, if gentamicin is used at higher dosages for bacterial biofilm removal, its ototoxicity and nephrotoxicity cannot be ignored. The antimicrobial mechanism of gentamicin is to mainly interact with bacterial ribosomes, inhibit bacterial protein synthesis, and disrupt the integrity of bacterial cell membranes (Appel and Neu, 1978). Curcumin is one of the most well-known antibacterial molecules from herbal medicine (Teow et al., 2016), and the synergistic anti-*S. aureus* effect with gentamicin is concentration-dependent, which is consistent with previous study (Teow and Ali, 2015). The major mechanism of CRM interact with *S. aureus* is to inhibit cytoplasmic division and bacterial proliferation *via* suppressing Z-loop formation (Rai et al., 2008), inhibit transcription of the mecA in MRSA to reverse resistance to β-lactam antibiotics (Mun et al., 2014), or bind to peptidoglycan in the cell wall to trigger cell wall damage and increase the membrane bidirectional permeability (Mun et al., 2014; Teow et al., 2016). Moreover, other studies also reported that *S. aureus* strain-dependence and no antagonistic effect were observed in its interaction with other antibiotics (Mun et al., 2013; Teow and Ali, 2015; Betts et al., 2016), which would probably also be related to the different biofilm formation condition, we adopted 1% human plasma, which has been demonstrated that fibronectin and its binding proteins are critical for MSSA and MRSA biofilms formation (Vergara-Irigaray et al., 2009; McCourt et al., 2014). Although the antimicrobial activity of isobavachalcone as a herbal extract has been investigated for the past decade (Wang M. et al., 2021), its anti-Staphylococcus activity has not been fully understood, a few studies reported its anti-*S. aureus* effect (Yin et al., 2004; Dzoyem et al., 2013; Cui et al., 2015; Faegheh et al., 2018). A recent study reported that the antibacterial activity of ISB against Gram-positive bacteria, specifically the *S. aureus* including MSSA and MRSA (with a MIC of 1.56 and 3.12 μg/ml, respectively) is associated with membrane disruption (Assis et al., 2022), hence ISB displayed no antibacterial effect on EPS in biofilm or Gram-negative species. Although the specific mechanism of antibacterial activity of this combination against *S. aureus* has not been fully investigated in this study, putting all the recent advances together, this process may be a synergistic multi-target action against *S. aureus* involving its cell wall integrity, membrane permeability, drug-resistant gene transcription, and protein biosynthesis, and cell wall and cell membrane could be their collaborative target.

To evaluate whether cocktail of Gentamicin + ISB + CRM can be used as a treatment for orthopedic implant-associated infections due to MRSA, a well-established femoral implant infection mouse model for simulating *in vivo* implant biofilm infection. Daily intraperitoneal administration of 20 mg/kg/day isobavachalcone, 20 mg/kg/day curcumin and 20 mg/kg/day gentamicin, can suppress progress of local infection. As we show *in vitro* and *in vivo* experiments, using gentamicin alone was difficult to suppress MRSA proliferation and local infection progression, but once added both ISB and CRM, the inhibitory and bactericidal effect of gentamicin against MRSA enhanced significantly. Although the antimicrobial effect of ISB or CRM has been investigated for the past decade (Wang M. et al., 2021), its anti-Staphylococcus activity has not been fully understood. Recent studies reported that the antibacterial effect of ISB and CRM are also associated with membrane disruption (Mun et al., 2014; Teow et al., 2016; Assis et al., 2022), specifically for the *S. aureus* including MSSA and MRSA (Assis et al., 2022) as aforementioned. So, the enhanced antimicrobial effect of gentamicin combined with ISB and CRM may result from their similar function of membrane disruption.

Cocktail treatment reduces inflammatory osteolysis and maintains microarchitecture of trabecular bone during orthopedic device-related MRSA infection in mice as well. Many studies have shown that *S. aureus* cell wall or biofilm components are the main causes of *S. aureus*-induced local inflammatory responses in the physiopathology of chronic osteomyelitis in ODRI, which is considered to be the classical animal model for studying the damage of bacterial products on bone structures in chronic osteomyelitis (Rochford et al., 2016). Therefore, we chose this animal model to study the pharmacological effects of isobavachalcone and curcumin on the tissue destruction induced by *S. aureus* biofilm. Microarchitecture of trabecular bone was found to be drastically deteriorated by μCT scanning and histopathology due to MRSA infection. However, treatment with isobavachalcone and curcumin significantly alleviated this process. The presence of local pathogenic microorganisms, such as *S. aureus*, can directly invade osteoblasts to form an intracellular infection (Tuchscherr et al., 2011; Jauregui et al., 2013; Hamza and Li, 2014), altering the autoimmune phenotype while decreasing osteoblast viability and osteogenic activity (Takayanagi, 2015). Previous studies have confirmed that RANKL/OPG signaling, TNF and TNF receptor superfamily, and the NF-kB signaling pathway play important roles in osteomyelitis in *S. aureus* infection (Lio et al., 2012; Krauss et al., 2019), and other studies also demonstrated that curcumin can inhibit NF-κB signaling pathway (Mohankumar et al., 2015; Kao et al., 2016). Isobavachalcone was reported capable of preventing osteoporosis by suppressing activation of ERK and NF-kappaB pathways of macrophages (Wang X. et al., 2021). It is probably that ISB combined with CRM treatment can protect bone microarchitecture under inflammatory microenvironment of device-related MRSA infection, attenuate osteolysis and avoid pathological fractures. These results validate the protection of cocktail of CRM + ISB, when administrated with Gentamicin, can attenuate inflammatory osteolysis induced by MRSA biofilm in ODRI.

Cocktail therapy also enhanced the reduction of MDSC M1 polarization in peri-implant tissue, suppression of MDSC amplification in peripheral blood *in vivo*. More interesting is that the bone density around the implant is increased in gentamicin combined with ISB or CRM group comparing to others according to μCT and histopathology. The number of MDSCs was significantly decreased, which suggested ISB and CRM may inhibit the expansion of MDSCs in *S. aureus* biofilm-induced myeloid cells and bidirectionally regulated the conversion of MSDC to polarized. Isobavachalcone was reported can prevent osteoporosis by suppressing M1 polarization of macrophages (Wang X. et al., 2021). A moderate M1-mediated inflammation is beneficial for tissue repair (Saqib et al., 2018), However, whether M1 phenotype plays a critical role in this osteogenesis process is still unclear. The persistence of bacteria stimuli in inflammatory osteogenesis may play another key factor in the tissue regeneration, because cell wall component lipoteichoic acid (LTA) has been reported to be beneficial for activation of osteoblast differentiation and Inhibition osteoclast activation in both *vivo* and *vitro* (Hu et al., 2020).

Notably, the fact that tissue destruction and regeneration in an inflammatory microenvironment during ODRI has always been a dilemma for surgeons. Without effective control of infection, there is no possibility of tissue regeneration. This study focus on investigating synergy of two small molecules both with anti-osteoporosis, anti-inflammation, and anti-bacteria characteristics. However, there are still several questions that need to be further addressed. First of all, although zero mortality was observed in this ODRI mouse model and no obvious hepatorenal toxicity of cocktail therapy compared to gentamicin alone for 1 month, their long-term toxicity and their pharmacokinetics *in vivo* remain unknown. Secondly, the major mechanism of enhancing gentamicin efficacy against *S. aureus* is not fully understood, especially whether new compounds would form in the interaction of the two molecules. And how will the promising cocktail work on a more mature and thicker 5-day biofilm? Thirdly, the mechanism of balance of bone resorption and promoting bone formation is unknown, and the mechanism of M1 reduction still needs further verification, for example, whether the suppression of *S. aureus* infection or M1 reduction plays an initial or fundamental role in this process. Last but not least, although most studies suggest that antimicrobial drugs maintain bone mass in osteomyelitis mainly by their direct and effective antimicrobial activity, and not indirectly by modulating immune cells to affect bone mass, whether cocktail therapy exhibits identical effect on immune cells with gold standard, for example, vancomycin, still needs to be studied.

In summary, these results suggest that the combination of isobavachalcone and curcumin can enhance the susceptibility of MRSA to gentamicin, thus promoting the eradication of MRSA biofilm. When administrated as cocktail *in vivo*, they can significantly modify local inflammation in orthopedic device-related infection and maintain trabecular bone microstructure while substantially eradication MRSA in ODRI. Although our current study did not reveal specific mechanism about the synergy of this cocktail of gentamicin, isobavachalcone and curcumin against *S. aureus*, their bone microarchitecture maintenance characteristic did provide us the insight and evidence for future potential topical application by incorporating the mixture of these two small molecules with conventional antibiotics, like gentamicin bone cement chain beads and antimicrobial biomaterials, etc. The combination of isobavachalcone and curcumin as adjuvants administrated together with gentamicin to significantly enhance its antimicrobial effect, which may serve as a new potential treatment strategy especially for MRSA-induced ODRI, to rationalize the use of high-level antibiotics and reduce the emergence of drug-resistant strains of bacteria.

## Data availability statement

The original contributions presented in the study are included in the article/Supplementary material, further inquiries can be directed to the corresponding authors.

## Ethics statement

The animal study was reviewed and approved by Animal Ethics Committee of the Guangzhou Huateng Education Incorporation and was acknowledged by the First Affiliated Hospital of Sun Yet-sen University.

## Author contributions

YC and XZ: conceptualization. YC, HH, FH, ZL, and XZ: design of research. YC and XZ: methodology. YC, HH, FH, BC, and XL: investigation. BC, BT, and TW: formal check and data analysis. CL and XZ: resources and funding acquisition. YC, HH, and ZL: writing the original manuscript. YC, HH, ZL, and XZ: major review and editing. All authors contributed to the article and approved the submitted version.

## Funding

This work was supported in part by National Natural Science Foundation of China (32071341, 32101062); Guangdong Basic and Applied Basic Research Foundation (2019A1515110005, 2020A1515110620, and 2022A1515012607); Chinese Postdoctoral Science Foundation (2021M693628); Science and Technology Program of Guangzhou (201804020011); the National Natural Science Foundation of Guangdong Province-Major Fundamental Research Fostering Program, China (2017A030308004); and the Fundamental Research Funds for the Central Universities, Sun Yat-sen University.

## Conflict of interest

The authors declare that the research was conducted in the absence of any commercial or financial relationships that could be construed as a potential conflict of interest.

The handling editor declared a shared parent affiliation with the authors at the time of review.

## Publisher’s note

All claims expressed in this article are solely those of the authors and do not necessarily represent those of their affiliated organizations, or those of the publisher, the editors and the reviewers. Any product that may be evaluated in this article, or claim that may be made by its manufacturer, is not guaranteed or endorsed by the publisher.

## References

[ref1] Appel G. B. Neu H. C. (1978). Gentamicin in 1978. Ann. Intern. Med. 89, 528–538. doi: 10.7326/0003-4819-89-4-528 358884

[ref2] Arciola C. R. Baldassarri L. Campoccia D. Creti R. Pirini V. Huebner J. (2008). Strong biofilm production, antibiotic multi-resistance and high gelE expression in epidemic clones of enterococcus faecalis from orthopaedic implant infections. Biomaterials 29, 580–586. doi: 10.1016/j.biomaterials.2007.10.008, PMID: 18029010

[ref3] Assis L. R. Theodoro R. D. S. Costa M. B. S. Nascentes J. A. S. Rocha M. D. D. Bessa M. A. S. (2022). Antibacterial activity of Isobavachalcone (IBC) is associated with membrane disruption. Membranes (Basel) 12:269. doi: 10.3390/membranes12030269, PMID: 35323743 PMC8950343

[ref4] Atashbeyk D. G. Khameneh B. Tafaghodi M. Fazly Bazzaz B. S. (2014). Eradication of methicillin-resistant *Staphylococcus aureus* infection by nanoliposomes loaded with gentamicin and oleic acid. Pharm. Biol. 52, 1423–1428. doi: 10.3109/13880209.2014.895018, PMID: 25026343

[ref5] Bernthal N. M. Stavrakis A. I. Billi F. Cho J. S. Kremen T. J. Simon S. I. (2010). A mouse model of post-arthroplasty *Staphylococcus aureus* joint infection to evaluate in vivo the efficacy of antimicrobial implant coatings. PLoS One 5:e12580. doi: 10.1371/journal.pone.0012580, PMID: 20830204 PMC2935351

[ref6] Betts J. W. Sharili A. S. La Ragione R. M. Wareham D. W. (2016). In vitro antibacterial activity of Curcumin-Polymyxin B combinations against multidrug-resistant bacteria associated with traumatic wound infections. J. Nat. Prod. 79, 1702–1706. doi: 10.1021/acs.jnatprod.6b00286, PMID: 27295561

[ref7] Biedenbach D. J. Moet G. J. Jones R. N. (2004). Occurrence and antimicrobial resistance pattern comparisons among bloodstream infection isolates from the SENTRY antimicrobial surveillance program (1997-2002). Diagn. Microbiol. Infect. Dis. 50, 59–69. doi: 10.1016/j.diagmicrobio.2004.05.003, PMID: 15380279

[ref8] Bimonte S. Barbieri A. Leongito M. Piccirillo M. Giudice A. Pivonello C. (2016). Curcumin AntiCancer studies in pancreatic cancer. Nutrients 8:433. doi: 10.3390/nu8070433, PMID: 27438851 PMC4963909

[ref9] Bjarnsholt T. Buhlin K. Dufrene Y. F. Gomelsky M. Moroni A. Ramstedt M. (2018). Biofilm formation – what we can learn from recent developments. J. Intern. Med. 284, 332–345. doi: 10.1111/joim.12782, PMID: 29856510 PMC6927207

[ref10] Campoccia D. Montanaro L. Moriarty T. F. Richards R. G. Ravaioli S. Arciola C. R. (2008). The selection of appropriate bacterial strains in preclinical evaluation of infection-resistant biomaterials. Int. J. Artif. Organs 31, 841–847. doi: 10.1177/039139880803100913, PMID: 18924097

[ref11] Costerton J. W. Stewart P. S. Greenberg E. P. (1999). Bacterial biofilms: a common cause of persistent infections. Science 284, 1318–1322. doi: 10.1126/science.284.5418.1318 10334980

[ref12] Cui Y. Taniguchi S. Kuroda T. Hatano T. (2015). Constituents of Psoralea corylifolia fruits and their effects on methicillin-resistant *Staphylococcus aureus*. Molecules 20, 12500–12511. doi: 10.3390/molecules200712500, PMID: 26184136 PMC6332258

[ref13] Cusack T. P. Ashley E. A. Ling C. L. Roberts T. Turner P. Wangrangsimakul T. (2019). Time to switch from CLSI to EUCAST? A southeast Asian perspective. Clin. Microbiol. Infect. 25, 782–785. doi: 10.1016/j.cmi.2019.03.016, PMID: 30922928 PMC6587905

[ref14] Daniel S. Limson J. L. Dairam A. Watkins G. M. Daya S. (2004). Through metal binding, curcumin protects against lead- and cadmium-induced lipid peroxidation in rat brain homogenates and against lead-induced tissue damage in rat brain. J. Inorg. Biochem. 98, 266–275. doi: 10.1016/j.jinorgbio.2003.10.014, PMID: 14729307

[ref15] Drago L. De Vecchi E. Mombelli B. Nicola L. Valli M. Gismondo M. R. (2001). Activity of levofloxacin and ciprofloxacin against urinary pathogens. J. Antimicrob. Chemother. 48, 37–45. doi: 10.1093/jac/48.1.37, PMID: 11418511

[ref16] Drago L. De Vecchi E. Nicola L. Gismondo M. R. (2007). In vitro evaluation of antibiotics’ combinations for empirical therapy of suspected methicillin resistant *Staphylococcus aureus* severe respiratory infections. BMC Infect. Dis. 7:111. doi: 10.1186/1471-2334-7-111, PMID: 17888153 PMC2025599

[ref17] Dzoyem J. P. Hamamoto H. Ngameni B. Ngadjui B. T. Sekimizu K. (2013). Antimicrobial action mechanism of flavonoids from Dorstenia species. Drug Discov. Ther. 7, 66–72. doi: 10.5582/ddt.2013.v7.2.66 23715504

[ref18] Espersen F. Frimodt-Moller N. Corneliussen L. Riber U. Rosdahl V. T. Skinhoj P. (1994). Effect of treatment with methicillin and gentamicin in a new experimental mouse model of foreign body infection. Antimicrob. Agents Chemother. 38, 2047–2053. doi: 10.1128/AAC.38.9.2047, PMID: 7811017 PMC284682

[ref19] Faegheh F. B K. Mehrdad I. Milad I. (2018). Antibacterial activity of flavonoids and their structure–activity relationship: an update review. Phytother. Res. 33, 13–40. doi: 10.1002/ptr.6208, PMID: 30346068

[ref20] Fagotti L. Tatka J. Salles M. J. C. Queiroz M. C. (2018). Risk factors and treatment options for failure of a two-stage exchange. Curr. Rev. Musculoskelet. Med. 11, 420–427. doi: 10.1007/s12178-018-9504-1, PMID: 29934884 PMC6105486

[ref21] Flemming H. C. Neu T. R. Wozniak D. J. (2007). The EPS matrix: the “house of biofilm cells”. J. Bacteriol. 189, 7945–7947. doi: 10.1128/JB.00858-07, PMID: 17675377 PMC2168682

[ref22] Gunes H. Gulen D. Mutlu R. Gumus A. Tas T. Topkaya A. E. (2016). Antibacterial effects of curcumin: an in vitro minimum inhibitory concentration study. Toxicol. Ind. Health 32, 246–250. doi: 10.1177/0748233713498458 24097361

[ref23] Hamza T. Li B. (2014). Differential responses of osteoblasts and macrophages upon *Staphylococcus aureus* infection. BMC Microbiol. 14:207. doi: 10.1186/s12866-014-0207-5, PMID: 25059520 PMC4116603

[ref24] Hirai J. Hagihara M. Kato H. Sakanashi D. Nishiyama N. Koizumi Y. (2016). Investigation on rifampicin administration from the standpoint of pharmacokinetics/pharmacodynamics in a neutropenic murine thigh infection model. J. Infect. Chemother. 22, 387–394. doi: 10.1016/j.jiac.2016.02.011, PMID: 27029221

[ref25] Hiramatsu K. Katayama Y. Yuzawa H. Ito T. (2002). Molecular genetics of methicillin-resistant *Staphylococcus aureus*. Int. J. Med. Microbiol. 292, 67–74. doi: 10.1078/1438-4221-00192 12195737

[ref26] Hu C. C. Chang C. H. Hsiao Y. M. Chang Y. Wu Y. Y. Ueng S. W. N. (2020). Lipoteichoic acid accelerates bone healing by enhancing osteoblast differentiation and inhibiting osteoclast activation in a mouse model of femoral defects. Int. J. Mol. Sci. 21:5550. doi: 10.3390/ijms21155550, PMID: 32756396 PMC7432397

[ref27] Huang H. Jin W. W. Huang M. Ji H. Capen D. E. Xia Y. (2020). Gentamicin-induced acute kidney injury in an animal model involves programmed necrosis of the collecting duct. J. Am. Soc. Nephrol. 31, 2097–2115. doi: 10.1681/ASN.2019020204, PMID: 32641397 PMC7461673

[ref28] Jauregui C. E. Mansell J. P. Jepson M. A. Jenkinson H. F. (2013). Differential interactions of Streptococcus gordonii and *Staphylococcus aureus* with cultured osteoblasts. Mol Oral Microbiol 28, 250–266. doi: 10.1111/omi.12022, PMID: 23413785

[ref29] Jin S. Meng C. He Y. Wang X. Zhang Q. Wang Z. (2020). Curcumin prevents osteocyte apoptosis by inhibiting M1-type macrophage polarization in mice model of glucocorticoid-associated osteonecrosis of the femoral head. J. Orthop. Res. 38, 2020–2030. doi: 10.1002/jor.24619, PMID: 32009245 PMC7496963

[ref30] John A. K. Baldoni D. Haschke M. Rentsch K. Schaerli P. Zimmerli W. (2009). Efficacy of daptomycin in implant-associated infection due to methicillin-resistant *Staphylococcus aureus*: importance of combination with rifampin. Antimicrob. Agents Chemother. 53, 2719–2724. doi: 10.1128/AAC.00047-09, PMID: 19364845 PMC2704655

[ref31] Jones M. Ying J. Huttner B. Evans M. Maw M. Nielson C. (2014). Relationships between the importation, transmission, and nosocomial infections of methicillin-resistant *Staphylococcus aureus*: an observational study of 112 veterans affairs medical centers. Clin. Infect. Dis. 58, 32–39. doi: 10.1093/cid/cit668, PMID: 24092798

[ref32] Kahlmeter G. Brown D. F. Goldstein F. W. MacGowan A. P. Mouton J. W. Odenholt I. (2006). European committee on antimicrobial susceptibility testing (EUCAST) technical notes on antimicrobial susceptibility testing. Clin. Microbiol. Infect. 12, 501–503. doi: 10.1111/j.1469-0691.2006.01454.x, PMID: 16700696

[ref33] Kaneko H. Nakaminami H. Ozawa K. Wajima T. Noguchi N. (2020). In vitro anti-biofilm effect of anti-methicillin-resistant *Staphylococcus aureus* (anti-MRSA) agents against the USA300 clone. J. Glob. Antimicrob. Resist. 24, 63–71. doi: 10.1016/j.jgar.2020.11.026, PMID: 33307275

[ref34] Kao N. J. Hu J. Y. Wu C. S. Kong Z. L. (2016). Curcumin represses the activity of inhibitor-kappaB kinase in dextran sulfate sodium-induced colitis by S-nitrosylation. Int. Immunopharmacol. 38, 1–7. doi: 10.1016/j.intimp.2016.05.015, PMID: 27233000

[ref35] Krauss J. L. Roper P. M. Ballard A. Shih C. C. Fitzpatrick J. A. J. Cassat J. E. (2019). *Staphylococcus aureus* infects osteoclasts and replicates Intracellularly. mBio 10:e02447-19. doi: 10.1128/mBio.02447-19, PMID: 31615966 PMC6794488

[ref36] Kunnumakkara A. B. Bordoloi D. Padmavathi G. Monisha J. Roy N. K. Prasad S. (2017). Curcumin, the golden nutraceutical: multitargeting for multiple chronic diseases. Br. J. Pharmacol. 174, 1325–1348. doi: 10.1111/bph.13621, PMID: 27638428 PMC5429333

[ref37] Lauderdale K. J. Malone C. L. Boles B. R. Morcuende J. Horswill A. R. (2010). Biofilm dispersal of community-associated methicillin-resistant *Staphylococcus aureus* on orthopedic implant material. J. Orthop. Res. 28, 55–61. doi: 10.1002/jor.20943, PMID: 19610092

[ref38] Lebeaux D. Ghigo J. M. Beloin C. (2014). Biofilm-related infections: bridging the gap between clinical management and fundamental aspects of recalcitrance toward antibiotics. Microbiol. Mol. Biol. Rev. 78, 510–543. doi: 10.1128/MMBR.00013-14, PMID: 25184564 PMC4187679

[ref39] Li Z. Knetsch M. (2018). Antibacterial strategies for wound dressing: preventing infection and stimulating healing. Curr. Pharm. Des. 24, 936–951. doi: 10.2174/1381612824666180213141109, PMID: 29436991

[ref40] Lio P. Paoletti N. Moni M. A. Atwell K. Merelli E. Viceconti M. (2012). Modelling osteomyelitis. BMC Bioinformatics 13:S12. doi: 10.1186/1471-2105-13-S14-S12, PMID: 23095605 PMC3439679

[ref41] Liu Y. W. An S. B. Yang T. Xiao Y. J. Wang L. Hu Y. H. (2019). Protection effect of Curcumin for macrophage-involved polyethylene Wear particle-induced inflammatory Osteolysis by increasing the cholesterol efflux. Med. Sci. Monit. 25, 10–20. doi: 10.12659/MSM.914197, PMID: 30599093 PMC6327781

[ref42] Lovati A. B. Drago L. Monti L. De Vecchi E. Previdi S. Banfi G. (2013). Diabetic mouse model of orthopaedic implant-related *Staphylococcus aureus* infection. PLoS One 8:e67628. doi: 10.1371/journal.pone.0067628, PMID: 23818985 PMC3688606

[ref43] McCourt J. O’Halloran D. P. McCarthy H. O’Gara J. P. Geoghegan J. A. (2014). Fibronectin-binding proteins are required for biofilm formation by community-associated methicillin-resistant *Staphylococcus aureus* strain LAC. FEMS Microbiol. Lett. 353, 157–164. doi: 10.1111/1574-6968.12424, PMID: 24628034

[ref44] Moghadamtousi S. Z. Kadir H. A. Hassandarvish P. Tajik H. Abubakar S. Zandi K. (2014). A review on antibacterial, antiviral, and antifungal activity of curcumin. Biomed. Res. Int. 2014:186864. doi: 10.1155/2014/186864, PMID: 24877064 PMC4022204

[ref45] Mohankumar K. Sridharan S. Pajaniradje S. Singh V. K. Ronsard L. Banerjea A. C. (2015). BDMC-A, an analog of curcumin, inhibits markers of invasion, angiogenesis, and metastasis in breast cancer cells via NF-kappaB pathway–a comparative study with curcumin. Biomed. Pharmacother. 74, 178–186. doi: 10.1016/j.biopha.2015.07.024, PMID: 26349982

[ref46] Molin S. Tolker-Nielsen T. (2003). Gene transfer occurs with enhanced efficiency in biofilms and induces enhanced stabilisation of the biofilm structure. Curr. Opin. Biotechnol. 14, 255–261. doi: 10.1016/s0958-1669(03)00036-3, PMID: 12849777

[ref47] Montanaro L. Speziale P. Campoccia D. Ravaioli S. Cangini I. Pietrocola G. (2011). Scenery of staphylococcus implant infections in orthopedics. Future Microbiol. 6, 1329–1349. doi: 10.2217/fmb.11.117, PMID: 22082292

[ref48] Mun S. H. Joung D. K. Kim Y. S. Kang O. H. Kim S. B. Seo Y. S. (2013). Synergistic antibacterial effect of curcumin against methicillin-resistant *Staphylococcus aureus*. Phytomedicine 20, 714–718. doi: 10.1016/j.phymed.2013.02.006, PMID: 23537748

[ref49] Mun S. H. Kim S. B. Kong R. Choi J. G. Kim Y. C. Shin D. W. (2014). Curcumin reverse methicillin resistance in *Staphylococcus aureus*. Molecules 19, 18283–18295. doi: 10.3390/molecules191118283, PMID: 25389660 PMC6271166

[ref50] Microbiology by Numbers. (2011). Microbiology by numbers. Nat. Rev. Microbiol. 9:628. doi: 10.1038/nrmicro2644 21961177

[ref51] Odds F. C. (2003). Synergy, antagonism, and what the chequerboard puts between them. J. Antimicrob. Chemother. 52:1. doi: 10.1093/jac/dkg301, PMID: 12805255

[ref52] Oikonomidis S. Altenrath L. Westermann L. Bredow J. Eysel P. Scheyerer M. J. (2020). Implant-associated infection of long-segment spinal instrumentation: a retrospective analysis of 46 consecutive patients. Asian Spine J. 15, 234–243. doi: 10.31616/asj.2019.0391, PMID: 32703924 PMC8055457

[ref53] Prince K. Kandi S. K. Sunny M. Kasturi M. Diwan S. R. (2019). Monocarbonyl Curcuminoids with improved stability as antibacterial agents against *Staphylococcus aureus* and their mechanistic studies. ACS Omega 4, 675–687. doi: 10.1021/acsomega.8b02625

[ref54] Rai D. Singh J. K. Roy N. Panda D. (2008). Curcumin inhibits FtsZ assembly: an attractive mechanism for its antibacterial activity. Biochem. J. 410, 147–155. doi: 10.1042/BJ20070891, PMID: 17953519

[ref55] Rao N. Ziran B. H. Lipsky B. A. (2011). Treating osteomyelitis: antibiotics and surgery. Plast. Reconstr. Surg. 127, 177S–187S. doi: 10.1097/PRS.0b013e3182001f0f 21200289

[ref56] Ribeiro M. Monteiro F. J. Ferraz M. P. (2012). Infection of orthopedic implants with emphasis on bacterial adhesion process and techniques used in studying bacterial-material interactions. Biomatter 2, 176–194. doi: 10.4161/biom.22905, PMID: 23507884 PMC3568104

[ref57] Rochford E. T. J. Sabate Bresco M. Zeiter S. Kluge K. Poulsson A. Ziegler M. (2016). Monitoring immune responses in a mouse model of fracture fixation with and without *Staphylococcus aureus* osteomyelitis. Bone 83, 82–92. doi: 10.1016/j.bone.2015.10.014, PMID: 26525592

[ref58] Sandikci A. S. Y A. F. Issa G. Basaran K. B. Dulger A. D. Buyukunal S. (2016). Antimicrobial effects of curcumin against *L. monocytogenes*, *S. aureus*, *S. Typhimurium* and *E. coli* O157: H7 pathogens in minced meat. Vet. Med. 61, 256–262. doi: 10.17221/8880-vetmed

[ref59] Saqib U. Sarkar S. Suk K. Mohammad O. Baig M. S. Savai R. (2018). Phytochemicals as modulators of M1-M2 macrophages in inflammation. Oncotarget 9, 17937–17950. doi: 10.18632/oncotarget.24788, PMID: 29707159 PMC5915167

[ref60] Sasidharan N. K. Sreekala S. R. Jacob J. Nambisan B. (2014). In vitro synergistic effect of curcumin in combination with third generation cephalosporins against bacteria associated with infectious diarrhea. Biomed. Res. Int. 2014:561456. doi: 10.1155/2014/561456, PMID: 24949457 PMC4052158

[ref61] Schimmel J. J. Horsting P. P. de Kleuver M. Wonders G. van Limbeek J. (2010). Risk factors for deep surgical site infections after spinal fusion. Eur. Spine J. 19, 1711–1719. doi: 10.1007/s00586-010-1421-y, PMID: 20445999 PMC2989231

[ref62] Slullitel P. A. Buttaro M. A. Greco G. Onativia J. I. Sanchez M. L. Mc Loughlin S. (2018). No lower bacterial adhesion for ceramics compared to other biomaterials: an in vitro analysis. Orthop. Traumatol. Surg. Res. 104, 439–443. doi: 10.1016/j.otsr.2018.03.003 29581066

[ref63] Song F. Liu J. Zhao W. Huang H. Hu D. Chen H. (2020). Synergistic effect of Eugenol and probiotic lactobacillus Plantarum Zs2058 against salmonella infection in C57bl/6 mice. Nutrients 12:1611. doi: 10.3390/nu12061611, PMID: 32486242 PMC7352263

[ref64] Tajbakhsh S. Mohammadi K. Deilami I. Zandi K. Fouladvand M. Ramedani E. (2008). Antibacterial activity of indium curcumin and indium diacetylcurcumin. Afr. J. Biotechnol. 7, 3832–3835. doi: 10.5897/AJB08.790

[ref65] Takayanagi H. (2015). Osteoimmunology in 2014: two-faced immunology-from osteogenesis to bone resorption. Nat. Rev. Rheumatol. 11, 74–76. doi: 10.1038/nrrheum.2014.219, PMID: 25561367

[ref66] Tallarida R. J. (2011). Quantitative methods for assessing drug synergism. Genes Cancer 2, 1003–1008. doi: 10.1177/1947601912440575, PMID: 22737266 PMC3379564

[ref67] Tawakoli P. N. Al-Ahmad A. Hoth-Hannig W. Hannig M. Hannig C. (2013). Comparison of different live/dead stainings for detection and quantification of adherent microorganisms in the initial oral biofilm. Clin. Oral Investig. 17, 841–850. doi: 10.1007/s00784-012-0792-3, PMID: 22821430

[ref68] Teow S. Y. Ali S. A. (2015). Synergistic antibacterial activity of Curcumin with antibiotics against *Staphylococcus aureus*. Pak. J. Pharm. Sci. 28, 2109–2114. 26639480

[ref69] Teow S. Y. Liew K. Ali S. A. Khoo A. S. Peh S. C. (2016). Antibacterial action of Curcumin against *Staphylococcus aureus*: a brief review. J. Trop. Med. 2016:2853045. doi: 10.1155/2016/2853045, PMID: 27956904 PMC5124450

[ref70] Tuchscherr L. Medina E. Hussain M. Volker W. Heitmann V. Niemann S. (2011). *Staphylococcus aureus* phenotype switching: an effective bacterial strategy to escape host immune response and establish a chronic infection. EMBO Mol. Med. 3, 129–141. doi: 10.1002/emmm.201000115, PMID: 21268281 PMC3395110

[ref71] Tyagi P. Singh M. Kumari H. Kumari A. Mukhopadhyay K. (2015). Bactericidal activity of curcumin I is associated with damaging of bacterial membrane. PLoS One 10:e0121313. doi: 10.1371/journal.pone.0121313, PMID: 25811596 PMC4374920

[ref72] Vanhommerig E. Moons P. Pirici D. Lammens C. Hernalsteens J. P. De Greve H. (2014). Comparison of biofilm formation between major clonal lineages of methicillin resistant *Staphylococcus aureus*. PLoS One 9:e104561. doi: 10.1371/journal.pone.0104561, PMID: 25105505 PMC4126748

[ref73] Vergara-Irigaray M. Valle J. Merino N. Latasa C. Garcia B. de Los R. (2009). Relevant role of fibronectin-binding proteins in *Staphylococcus aureus* biofilm-associated foreign-body infections. Infect. Immun. 77, 3978–3991. doi: 10.1128/IAI.00616-09, PMID: 19581398 PMC2738049

[ref74] Wang X. Ji Q. Hu W. Zhang Z. Hu F. Cao S. (2021). Isobavachalcone prevents osteoporosis by suppressing activation of ERK and NF-kappaB pathways and M1 polarization of macrophages. Int. Immunopharmacol. 94:107370. doi: 10.1016/j.intimp.2021.107370, PMID: 33640858

[ref75] Wang M. Lin L. Lu J. J. Chen X. (2021). Pharmacological review of isobavachalcone, a naturally occurring chalcone. Pharmacol. Res. 165:105483. doi: 10.1016/j.phrs.2021.105483, PMID: 33577976

[ref76] Wang J. Zhou X. Li W. Deng X. Deng Y. Niu X. (2016). Curcumin protects mice from *Staphylococcus aureus* pneumonia by interfering with the self-assembly process of alpha-hemolysin. Sci. Rep. 6:28254. doi: 10.1038/srep28254, PMID: 27345357 PMC4921848

[ref77] Xu W. Dong S. Han Y. Li S. Liu Y. (2018). Hydrogels as antibacterial biomaterials. Curr. Pharm. Des. 24, 843–854. doi: 10.2174/1381612824666180213122953 29436994

[ref78] Yin S. Fan C. Q. Wang Y. Dong L. Yue J. M. (2004). Antibacterial prenylflavone derivatives from *Psoralea corylifolia*, and their structure-activity relationship study. Bioorg. Med. Chem. 12, 4387–4392. doi: 10.1016/j.bmc.2004.06.014, PMID: 15265490

