# Supplementary material for: Cocktail of isobavachalcone and curcumin enhance eradication of Staphylococcus aureus biofilm from orthopedic implants by gentamicin and alleviate inflammatory osteolysis
Source: Front Microbiol. 2022 Sep 23;13:958132. doi: 10.3389/fmicb.2022.958132 (PMC9537636; doi:10.3389/fmicb.2022.958132)
Supplement: Supplementary file 1 [file Table_1.DOCX]

Supplementary Material

# Supplementary Tables

**Supplementary Table 1.** MIC/MBC/MBEC of tested molecules in preliminary study

| Compounds against S. aureus | | | | | | | | | | | | |
| --- | --- | --- | --- | --- | --- | --- | --- | --- | --- | --- | --- | --- |
| No. | Bacteria Strain | Abb. | MIC*  (μg/mL) | MBC*  (μg/mL) | MBEC*  (μg/mL) |  | No. | Compound | Abb. | MIC*  (μg/mL) | MBC*  (μg/mL) | MBEC*  (μg/mL) |
| 1 | S. aureus JAR | ISB | 6.25 | 12.5 | ＞25 |  | 11 | S. aureus JAR | CRM | 500 | ＞2000 | - |
| 2 | ATCC29923 | ISB | 6.25 | 12.5 | ＞25 |  | 12 | ATCC29923 | CRM | 500 | ＞2000 | - |
| 3 | MSSA 2222 | ISB | 6.25 | 12.5 | ＞25 |  | 13 | MSSA 2222 | CRM | 500 | ＞2000 | - |
| 4 | MSSA2557 | ISB | 6.25 | 12.5 | ＞25 |  | 14 | MSSA2557 | CRM | 500 | ＞2000 | - |
| 5 | MSSA2039 | ISB | 6.25 | 12.5 | ＞25 |  | 15 | MSSA2039 | CRM | 500 | ＞2000 | - |
| 6 | MSSA 2031 | ISB | 6.25 | 12.5 | ＞25 |  | 16 | MSSA 2031 | CRM | 1000 | ＞2000 | - |
| 7 | USA300 | ISB | 12.5 | 25 | - |  | 17 | USA300 | CRM | 2000 | ＞2000 | - |
| 8 | MRSA2435 | ISB | 12.5 | 25 | - |  | 18 | MRSA2435 | CRM | 2000 | ＞2000 | - |
| 9 | MRSA2027 | ISB | 12.5 | 25 | - |  | 19 | MRSA2027 | CRM | 2000 | ＞2000 | - |
| Ctrl | DMSO | DMSO | 25% | 50% | 50% |  |  |  |  |  |  |  |
|  |  |  |  |  |  |  |  |  |  |  |  |  |
| Antimicrobials against MSSA ATCC 25923 | | | | | | | | | | | | |
| No. | antibiotic | Abb. | MIC*  (μg/ml) | MBC*  (μg/ml) | MBEC*  (μg/ml) |  | # | antibiotic | Abb. | MIC*  (μg/ml) | MBC*  (μg/ml) | MBEC*  (μg/ml) |
| 1 | Daptomycin | DAP | 1 | 2 | ＞1024 |  | 6 | Gentamycin | GEN | 1 | 2~4 | ＞1024 |
| 2 | Daptomycin 50 | DAP 50 | 0.5 | 1 | 128 |  | 7 | Clindamycin | CLI | 0.125 | 0.5 | ＞1024 |
| 3 | Daptomycin 100 | DAP 100 | 0.25 | 1 | 128 |  | 8 | Fosfomycin | FOS | 4 | 4 | ＞1024 |
| 4 | Rifampacin | RIF | 0.008 | ＞0.5 | ＞1024 |  | 9 | Vancomycin | VAN | 1 | 2 | ＞1024 |
| 5 | Levofloxacin | LEV | 0.25 | 0.25 | ＞1024 |  | 10 | Trimethoprim-Sulfamethoxazole | TMP-SMX(TS) | 0.125 | 0.25 | ＞1024 |
|  |  |  |  |  |  |  |  |  |  |  |  |  |

MIC: minimum inhibitory concentration; MBC: minimum bactericidal concentration; MBEC: minimum biofilm eradication concentration; Daptomycin 50 and Daptomycin 100 refer to daptomycin containing 50 μg/ml and 100 μg/ml Ca2+, respectively. ISB: isobavachalcone; CRM: curcumin. *: “>” represent MICs/MBCs/MBECs are not acquired at the maximum tested concentration; #: “-“ represents the no MBEC was acquired or cannot be evaluated. DMSO concentration is presented in volume ratio as control.

## Supplementary Figures


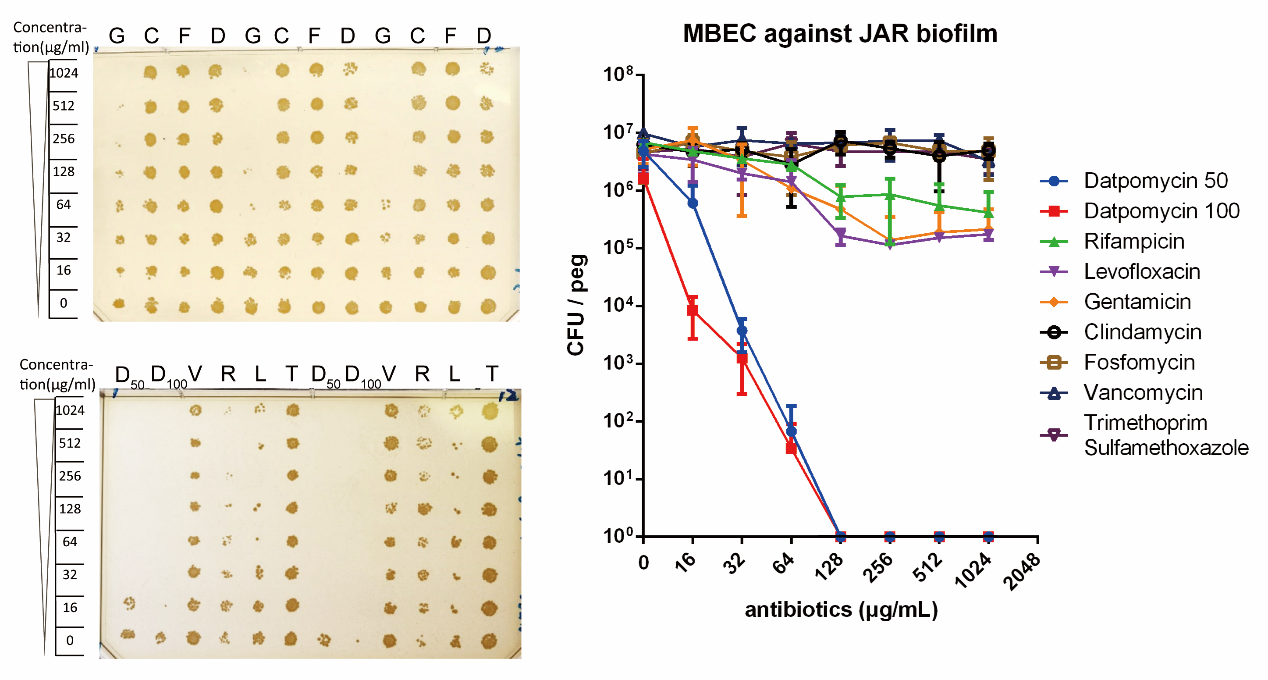


**Supplementary Figure 1.** MBEC assay of antibiotics against S. aureus JAR biofilm. A) Colony formation in single-well TSA agar plates represents the amount of residual bacteria in biofilm after 24-h antibiotics challenge. B) Viable cells remaining on S. aureus JAR biofilm challenge by antibiotics for 24 h. N= 6/per group, all data are presented as mean ± SD. D: daptomycin; D50: daptomycin 50μg/ml Ca2+; D100: daptomycin 100μg/ml Ca2+; G: gentamycin; C: clindamycin; F: Fosfomycin; V: vancomycin; R: rifampicin; T: Trimethoprim Sulfamethoxazole.
